# Comprehensive genetic dissection of wood properties in a widely-grown tropical tree: *Eucalyptus*

**DOI:** 10.1186/1471-2164-12-301

**Published:** 2011-06-08

**Authors:** Jean-Marc Gion, Audrey Carouché, Sylvie Deweer, Franck Bedon, Frédérique Pichavant, Jean-Paul Charpentier, Henri Baillères, Philippe Rozenberg, Victor Carocha, Nina Ognouabi, Daniel Verhaegen, Jacqueline Grima-Pettenati, Philippe Vigneron, Christophe Plomion

**Affiliations:** 1 CIRAD, Department of Biological System, Research Unit "Genetic improvement and adaptation of mediterranean and tropical plants" TA A-108/C, Campus International de Baillarguet, 34398 Montpellier Cedex, France; 2 USBB - Institut du Pin, 351 cours de la libération, 33405 TALENCE Cedex, France; 3 INRA, Unité d'Amélioration, Génétique et Physiologie Forestières, Olivet 45166, France; 4 CIRAD, Department of Tropical Production system and Process Performance, Research Unit "Processing and promotion of Tropical Woods" 73 rue J-F Breton - TA B-40/16 - 34398 Montpellier Cedex 5, France; 5 RAIZ - Forestry & Paper Research Institute, Qta S. Francisco, Apartado 15, Eixo, 3801-501 Aveiro, Portugal; 6 CRDPI, BP 1291, Pointe Noire, République du Congo; 7 UMR UPS/CNRS 5546, Pôle de Biotechnologies Végétales, 24 chemin de Borde Rouge, BP42617, Auzeville Tolosane, 31326 Castanet Tolosan, France; 8 INRA, UMR 1202 BIOGECO, 69 route d'Arcachon, 33612 Cestas Cedex, France

**Keywords:** Wood properties, QTL, genetic mapping, candidate genes, *Eucalyptus*

## Abstract

**Background:**

*Eucalyptus *is an important genus in industrial plantations throughout the world and is grown for use as timber, pulp, paper and charcoal. Several breeding programmes have been launched worldwide to concomitantly improve growth performance and wood properties (WPs). In this study, an interspecific cross between *Eucalyptus urophylla *and *E. grandis *was used to identify major genomic regions (Quantitative Trait Loci, QTL) controlling the variability of WPs.

**Results:**

Linkage maps were generated for both parent species. A total of 117 QTLs were detected for a series of wood and end-use related traits, including chemical, technological, physical, mechanical and anatomical properties. The QTLs were mainly clustered into five linkage groups. In terms of distribution of QTL effects, our result agrees with the typical L-shape reported in most QTL studies, i.e. most WP QTLs had limited effects and only a few (13) had major effects (phenotypic variance explained > 15%). The co-locations of QTLs for different WPs as well as QTLs and candidate genes are discussed in terms of phenotypic correlations between traits, and of the function of the candidate genes. The major wood property QTL harbours a gene encoding a Cinnamoyl CoA reductase (CCR), a structural enzyme of the monolignol-specific biosynthesis pathway.

**Conclusions:**

Given the number of traits analysed, this study provides a comprehensive understanding of the genetic architecture of wood properties in this *Eucalyptus *full-sib pedigree. At the dawn of *Eucalyptus *genome sequence, it will provide a framework to identify the nature of genes underlying these important quantitative traits.

## Background

*Eucalyptus *is the main hardwood genus used in intensively managed forest tree plantations. Because of its high productivity on marginal sites, high fibre content and usefulness in producing a wide range of forest products, *Eucalyptus *has been widely planted in subtropical, tropical and temperate regions of the world, accounting for 8% of the planted forest area [1]. Intensive silvicultural practices and traditional genetic improvement have both contributed to improve the productivity of wood biomass [2]. Wood properties (WPs) have also become mandatory traits in breeding programmes for the development of improved varieties for pulp and paper, energy wood or timber production.

WPs vary greatly between species, within species and within a tree (reviewed by Plomion et al. [3]) and change with age (reviewed by Raymond [4]). They can be classified in five categories: i) mechanical properties in response to applied forces (e.g. longitudinal growth strain, modulus of elasticity, strength), ii) technological characteristics, which are the consequences of the mechanical state of the tree (e.g. splitting index), iii) physical characteristics, corresponding to the natural characteristics of wood which affect mechanical properties (e.g. wood density, shrinkage), iv) anatomical characteristics (e.g. fibre length, fibre thickness, coarseness), and v) traits related to chemical composition (e.g. cellulose and lignin content, lignin composition). A key constraint to higher-value uses of some *Eucalyptus *species used for timber production is their high internal stresses in the stem [5-7]. The release of growth stresses during cross-cutting may cause radial cracks at the log end. This "end-splitting" phenomenon increases with time according to an asymptotic curve. The extent of this degrade can be explained by the high level of growth stresses, low wood transverse strength and gradients of mechanical properties [8]. Previous studies indicated that growth stresses were not correlated with growth or other WPs [9-11]. The chemical composition (cellulose, hemi-celluloses, lignins, flavonoids, tannins, etc.) of wood also greatly influences the properties and performance of pulp, paper and charcoal. Different authors reported strong correlations between pulp yield and cellulose content in *E. urophylla *[12] or lignin content in *E. globulus *[13]. Another study suggested that the lower kraft pulp yield of *E. viminalis *can, to a large extent, be attributed to the higher soluble and insoluble tannin contents of the wood [14]. Interestingly, lignin content has been reported to be negatively correlated with longitudinal growth strain in *Eucalyptus *hybrids [8], suggesting a relationship between micro (chemical composition) and macro (mechanical properties) characteristics of wood.

The genetic determinism of WPs has been fairly well studied in *Eucalyptus *[4]. In *E. globulus*, Apiolaza et al. [15] reported moderate to high heritability for several WPs without any significant genetic correlation with growth, except between microfibril angle and trunk diameter (-0.86). In *E. grandis*, no significant correlation was found between wood density and growth [16]. In *E. urophylla*, a narrow sense heritability of 0.5 was estimated for cellulose content with no correlation with growth [12]. Silva *et al*. [17] found a significant genetic correlation between wood density (a highly heritable trait) and growth diameter in *E. globulus*. Other authors reported a significant correlation between WPs, *e.g*. microfibril angle and basic density (-0.70) and pulp yield and cellulose content (0.82) [15]. Together, these results suggest: i) a higher heritability for WPs than growth-related traits, ii) some form of pleiotropic relationships between different WPs or close linkage between the genes controlling these traits, and iii) an inconsistency regarding the extent of genetic correlations between WPs and growth. Despite attempts to describe the number, location, effects and nature of the genes underlying these quantitative traits, up to now, very little effort has been made toward a more comprehensive analysis of the genetic architecture of WPs taking into account in a single experiment mechanical, physical, technological, anatomical and chemical properties.

Investigating the genetic basis of multi-scale WPs has become a major issue while the sequence of the *Eucalyptus *genome is nearing completion [2,18]. Dissecting the genetic architecture of such complex traits can be addressed by QTL mapping [19]. In *Eucalyptus*, several QTL studies have focused on wood density [20-22]. Barros et al. [23] identified molecular markers linked to wood splitting in open-pollinated *E. grandis*. Thamarus et al. [24] published the first significant results on the genetic architecture of WPs using high throughput phenotyping methods to detect QTLs for fibre length, cellulose, pulp yield and microfibril angle (MFA) in *E. globulu*s. Three QTLs (wood-fibre, wood density and pulp yield) were detected across two half-sib pedigrees indicating a stable effect of the genetic control across different genetic backgrounds. Rocha et al. [25] found co-located QTLs for pulp yield and lignin content in an interspecific cross *E. grandis *× *E. urophylla*. Similar linkages were reported between pulp yield and cellulose content in *E. globulus *[24]. More recently, Freeman et al. [26] discovered several genomic regions of *E. globulus *affecting physical (density) and chemical wood properties, e.g. co-located QTLs for lignin content, cellulose content and pulp yield. Finally, Thumma et al. [27] identified QTLs for density, MFA and wood chemical components in *E. nitens*.

The availability of *Eucalyptus *sequences in public databases (reviewed in Myburg et al. [28]) makes it possible to propose functional candidate genes *a priori *involved in the genetic control of the traits concerned, e.g. structural and regulatory genes involved in lignification for traits related to lignin content and lignin quality. Differentiating secondary xylem libraries [29] and high-throughput transcriptome sequencing [30] are also useful sources of functional molecular markers to decipher the nature of QTLs for WPs. Some co-locations between QTLs for wood quality related traits and functional candidate genes have already been reported: e.g. between a transcription factor (EgMYB2) and a QTL for lignin content in *E. grandis *[31], between a vascular-expressed Rac-like small GTPase (EgROP1) and lignin composition and fibre morphology in *E. urophylla *[32], between the gene encoding a cinnamoyl-CoA reductase (CCR) and a QTL for cellulose content in *E. globulus *[24], and between CCR and a QTL for MFA in *E. nitens *[27].

In this context, the objectives of this study were two-fold: i) to perform an extensive QTL analysis for a large range of WPs in an interspecific cross of *Eucalyptus *in order to dissect the genetic architecture of these complex multi-scale traits, ii) to identify positional candidate genes co-locating with WP-QTLs, as the basis of future positional cloning of major QTLs.

## Methods

### Genetic material and DNA extraction

An interspecific hybrid progeny (F1 outbreed) of *E. urophylla *and *E. grandis *was used to dissect wood properties into their Mendelian inherited components (QTLs). This progeny was also used by Verhaegen and Plomion [33], Verhaegen et al. [21] and Gion et al. [34] in previous linkage and QTL mapping studies. A total of 201 full-sibs planted in 1992 were felled at 59 months old. Genomic DNA was extracted from dried leaves according to Doyle and Doyle [35] with minor modifications described by Verhaegen and Plomion [33].

### Phenotypic assessment

**Figure 1:**
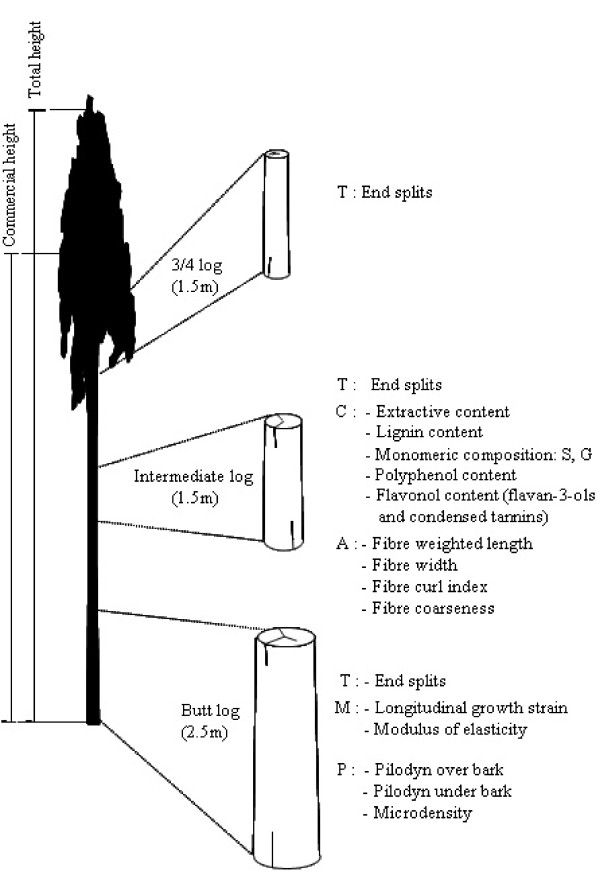
**Wood quality traits measured in different logs cut from the stem according to five classes: Technological (T), Mechanical (M), Chemical (C), Fibre (A) and Physical (P) properties**.

Total height (Ht59) and trunk circumference at 1.3 m (Cir59) were measured once the trees were cut down at 59 months old. Three logs were cut from each tree, corresponding to a butt log (2.5 m in length), an intermediate log (1.5 m in length) taken at half the commercial height (i.e. height measured at 8 cm diameter), and a third log (1.5 m in length) sampled at three-quarters of the commercial height. WPs were measured at different levels as shown in Figure 1.

#### Chemical properties

A 7-cm disk was sampled in the middle of the intermediate log and crushed into wood powder (40 mesh). Four types of traits were measured: i) lignin content, ii) lignin composition, iii) polyphenol, and iv) flavanol contents. Klason lignin was measured according to Tappi T222 om-83 and the modified procedure of Effland [36]. Lignin monomers were characterized by thioacidolysis, an efficient procedure to estimate the amount of Syringyl (S), Guaiacyl (G) and Hydroxyphenyl (H) units [37]. Total polyphenols (Poly) and flavanols (flavan-3-ols and condensed tannins, Fla) were measured using 50 mg of *Eucalyptus *dry wood meal, after two successive extractions with 2 mL of 80% methanol. The mixture was sonicated for 30 min before being centrifuged at 18,000 g for 10 min. Supernatants from both extractions were pooled. All the steps were carried out at 4°C. For Poly quantification, 500 μL of supernatants were dried under vacuum using a "Speed-Vac" system (Savant Instrument) and suspended in 500 μL ultra-pure water. Poly content was estimated by colorimetric assay based on oxidation-reduction reactions with Folin-Ciocalteu reagent [38,39]. 100 μL of phenolic extract, 500 μL of Folin-Ciocalteu reagent (Sigma, diluted 10 times in ultra-pure water) and 400 μL of NaCO_3 _75 g.L^-1 ^were successively mixed and incubated for 5 min at 40°C. Absorbance at 735 nm was measured spectrophotometrically and the results expressed in mg equivalent of Gallic acid per gram of dry weight. Gallic acid aqueous solutions (0-20 μg.mL^-1^) were used for calibration. For Fla quantification, 1 mL of supernatant was dried and suspended in 100 μL methanol. Fla was quantified by a colorimetric method using 4-dimethylamino-cinnamaldehyde (DMACA - Sigma) as chemical reagent [40]: 100 μL of phenolic extract, 880 μL of methanol and 20 μL of DMACA solution (100 mg DMACA in 10 mL of 1.5 M methanolic sulfuric acid) were mixed and incubated for 2 h at room temperature. Fla was determined spectrophotometrically at 630 nm using a standard curve based on catechin (0-15 μg.mL^-1^) and expressed in mg equivalent of catechin per gram of dry weight.

#### Assessment of growth stresses and related degrade

i) One week after felling, the circumference in the middle of the 2.5 mg butt log was divided into four to eight equal sectors depending on the diameter. At the middle of each sector, the "single hole" drilling method of Fournier et al. [41] was used to estimate the longitudinal growth strain (LGS) close to the trunk surface. This method measures the relative displacement of two pins inserted in the wood after releasing longitudinal growth stress in the outermost wood layer. A positive displacement resulting from shortening of the fibres, corresponds to the release of a tensile stress. Finally the mean of the four or eight measurements (according to trunk diameter) was used for QTL analysis.

ii) On each butt log, a splitting index (SI) was used to assess the intensity of the log end split. The index is calculated as follows:

Three SI measurements were made one day (SId), one week (SIw) and one month (SIm) after the trees were felled. The average of basal and terminal log ends was used for QTL analysis for these three measurements.

#### Longitudinal modulus of elasticity (MOE)

Trees were cut into logs of 400 mm long at 1.30 m above the ground. From each log, one slab was taken out along the diameter in a north-south direction. Each slab was divided into specimens of constant dimension from the bark towards the pith. Three to eight specimens 400 mm long (longitudinal direction) × 20 mm thick (radial direction) × 40 mm wide (tangential direction) were prepared from each tree. A total of 808 specimens were available for the measurement of the longitudinal modulus of elasticity (MOE) which was measured on specimens at green condition using the non-destructive method of Brancheriau and Baillères [42] based on spectral analysis of natural bending vibrations in the tangential-longitudinal plane. This technique gave a relative accuracy estimated at 6% [42]. The measured values of E were interpreted as the averaged values of MOE of concentric wood tissues included in each sample.

To facilitate the analysis of these data and provide a better understanding of the variability of wood, a method was developed to improve handling of within-tree variation of wood properties using a few discrete data averaged on large sized specimens [8]. Based on a non-deterministic and non-parametric technique for describing continuous variation in wood characteristics, this is a new approach for statistical analyses and graphical presentation that does not use arbitrarily chosen mathematical models. Local and tree averaged E property increases with cambial age according to a sigmoid profile that can be described by three characteristic parameters:

• E_p_: MOE at pith,

• E_30_: average MOE at 30 months cambial age

• E_2i_: average MOE at 2 times inflection point of cambial age *vs*. MOE

#### Fibre properties

Fibre properties were obtained from a disk sampled in the middle of the intermediate log. Fibre characteristics were determined using PQM 1000 apparatus. Measurements were made on 2-g samples of pulp. Three properties were estimated: fibre weighted length (FWL), which reduces the effect of small fibre fragments, coarseness (Coar) a measure of mass per unit length of fibre, and curl index (Curl) measured as [real length/projected length-1] × 100 which is an assessment of the straightness of the fibre.

#### Wood density

Wood density was indirectly estimated at breast height before felling, using the Pilodyn^® ^as described by Verhaegen et al. [21]. The penetration depth of the pilodyn pin (measured in millimetres, PIL) is an indirect estimation of wood density (WD), which is negatively correlated with WD. Five measurement dates were available, *i.e*. at month 14, 26, 38 (data already published, see [21]), and at month 52, and 59 (this study) after planting. At 59 months, one more pilodyn measurement was made under bark. Microdensity (μD) profiles were also obtained on a disk cut at breast height, using the indirect X-ray method developed by Polge [43] and improved since then in different laboratories around the world [44]. The microdensity profile measures the radial variation of wood density at a fine scale (25 μm), from pith to bark. In this study we used the average of the complete microdensity profile (i.e. mean microdensity) as an estimate of trunk density.

### Molecular markers

#### Choice of candidate genes

These genes included: i) eight functional candidate genes involved in lignin biosynthesis, including genes of the common phenylpropanoid pathway [caffeic acid 3-0-methyltransferase (COMTA and B), caffeoyl CoA 3-O-methyltransferase (CCoAOMT), 4-coumarate CoA ligase (4 CL), phenylalanine ammonia-lyase (PAL) and p-coumaroyl-CoA-3-hydroxylase (C3H)] and genes involved in the 'lignin specific' pathway [cinnamoyl-CoA-reductase (CCR) and cinnamyl alcohol dehydrogenase (CAD)], ii) two regulatory genes putatively involved in the regulation of these structural genes: EgMYB2, a member of the R2R3 MYB family of transcription factors [31] and a Rac-like small GTPase involved in cell differentiation during secondary xylem formation (EgROP1; [32]), and iii) 84 expressional candidate genes selected from a list of genes preferentially expressed in *Eucalyptus *wood forming tissue compared to leaf [29].

#### Other molecular markers

Molecular markers included: two symbiosis regulated genes (EgHypar, [45]; and EgTubA1, [46]), one paralogue of the leafy gene family (LFY1, [47,48]), one member of the Chalcone synthase gene family (CHS), three genes of the methionine pathway (SAH, SAMS, HMT from Kirst et al. [49]), 20 simple sequence repeats (SSRs) from Brondani et al. [50], and 147 Random Amplified Polymorphic DNA (RAPD) markers from Verhaegen et al. [21].

### Polymorphism detection and linkage mapping

Gene polymorphism was revealed using the SSCP (single strand conformation polymorphism, [51]) technique as described in Gion et al. [34]. SSR genotyping was performed using LI-COR automated sequencers as described in Mariette et al. [52]. A map was constructed for each parent of the cross following the two-way pseudo-testcross mapping strategy [53], using Mapmaker/exp ver. 3.0 [54]. In brief, loci were assigned to linkage groups (LG) with a minimum LOD score of 3.0 and a maximum Kosambi distance of 40 centiMorgans (cM). The order of the markers was approximated using the FIRST ORDER function of Mapmaker. Framework maps were then constructed by comparing the likelihood of all permutations of all adjacent triplets using the RIPPLE function. Individual markers were dropped from each linkage group until a marker sequence was obtained that had an order at least 1,000 times better than alternative orders (*i.e*., log likelihood difference ≥3). The markers that were dropped were placed on the framework map as accessory markers and located near the closest framework markers.

### QTL mapping

For QTL detection, we used the Multiple Interval Mapping (MIM) procedure [55] implemented in MultiQTL V2.6 (Haifa, Israel, 2005; [http://www.multiqtl.com/]). MIM allows individual QTLs to be detected independently of background noise and of previously detected QTLs.

Multi-QTL uses statistical models to estimate the significance of a QTL, where models with one and two QTLs per LG are proposed. The single QTL model tests the null hypothesis that there is a single QTL on the LG against the absence of QTL (H_1 _vs. H_0_). The two-linked QTL model tests the hypothesis of the presence of two QTLs on the LG: (1) against the hypothesis of the absence of QTL (H_2 _vs. H_0_) and (2) against the hypothesis of the presence of a single QTL (H_2 _vs. H_1_). The MIM procedure of Multi-QTL allows the appropriate model to be attributed to each LG. QTLs were detected in a two-step procedure: first a MIM was performed using a two-linked QTL model on each LG, then a MIM with either a two-linked QTL model on LGs harbouring two linked QTL from the first step, or a single QTL model on the other LGs, was performed.

Empirical statistical significance thresholds (p), determined by 1,000 permutations of the dataset, were used to declare the presence of a QTL [56]. In our study, QTLs were declared significant using a type I error rate of 10% at the genome level. Taking into account the mean number of markers per linkage group, this threshold roughly corresponds to a 1% error rate at the chromosome level [57]. Therefore, instead of the 5% default chromosome level detection rate implemented in MultiQTL, we used a more stringent statistical threshold for MIM analyses. This contrasts with the recent use of multiQTL [57-59] where MIM analysis was performed with the default detection threshold (5% at the chromosome level) and where a genome-wise p-value was computed *a posteriori *and separately based on the number of mapped loci. Such a procedure should be avoided because it does not take into account the proper type I error rate in the detection step.

The empirical confidence interval at 95% for the QTL position and for the allelic effect was calculated using bootstrap analysis [60] with 5,000 resamplings. We defined the "position range" of a QTL as the range between the position calculated from the original dataset (P) and the position calculated from bootstrap analysis (PBS). The confidence interval of the QTL position was calculated relative to the PBS.

The allelic substitution effect of the QTL and the percentage of explained phenotypic variance (PEV) were calculated using the MIM procedure. For each significant QTL, the proportion of phenotypic variance explained was estimated as follows: i/for single-trait QTL analysis:

PEV = ¼ d^2^/σ ^2 ^_ph_, where d is the substitution allelic effect and σ^2 ^is the phenotypic variance; ii/for two-linked QTL effect, PEV = [(d_1_+d_2_)^2^/4 - rd_1_d_2 _+ 4(1-r)rε^2^]/(σ^2 ^_ph_), where d_1 _and d_2 _are the effects of the two QTLs, ε is the epistasis and r is the rate of recombination between the mapped QTLs. Charts of linkage maps and QTL positions were drawn using MapChart version 2.2 [61].

### Probabilities of co-location of QTL/QTL and QTL/Candidate Genes

To test whether the overlap of QTLs for two traits was due to chance alone, we calculated the probability of co-occurrences of QTLs in the same genomic region under the null hypothesis of random distribution of QTLs, (adapted from Scotti-Saintagne et al. [57]). For this analysis, the genome was subdivided into n_1 _intervals. Each interval corresponded to the mean distance between the position of the highest LOD score from the composite interval mapping and the position of the mean value for maximum LOD score after bootstrap analysis. If n_3 _is the number of QTLs for the trait exhibiting the largest number of QTLs, n_4 _the number of QTLs for the second trait, and n_2 _the number of QTLs shared by the two traits, then the probability of having n_2 _intervals in common is given by Lin et al. [62]:

## Results

### Variability and correlation between wood properties

A total of 201 full-sibs of an interspecific hybrid progeny between *E. urophylla *and *E. grandis *were felled at 59 months and used to dissect wood properties. The statistics for each trait and their phenotypic correlations are listed in Tables 1 and 2. Coefficients of phenotypic variation (CPV) were rather low in the family tested (3 to 18%), except for five traits (SId, SIw, SIm, Poly and Fla) for which CPV varied between 38% and 59%. As commonly reported for hardwoods and eucalypts in particular [63], S units (2504 mol.g^-1 ^of lignin on average) were more abundant that G units (624 mol.g^-1 ^of lignin on average), S also presented a higher level of variability. No significant correlation was observed between traits belonging to different categories of WPs, except between density and modulus of elasticity (positive correlation). The correlation coefficient was high between S and G (0.6) and moderate between Klason lignin content (Klas) and the S/G ratio (-0.29), suggesting that trees with a higher lignin content had lower S unit content. Poly and Fla contents were highly correlated (0.64). Fibre related traits had lower coefficients of variation than the other traits and were highly correlated with each other, either positively (WFL and Coar 0.73) or negatively (WFL and Curl -0.61, Coar and Curl -0.50). Slipping indexes were positively correlated (ranging from 0.38 to 0.74) and showed moderate correlation with LGS (0.34-0.41). A positive and strong age-age correlation was observed for Pil from age 14 to age 59 months and each Pil measurement over bark was strongly positively correlated with Pil under bark at 59 months. As expected, all Pil measurements were correlated (-0.35 to -0.55) with mean microdensity. In addition, positive correlations were detected between growth (height and circumference measured at 59 months) on the one hand, and mechanical (LGS) and technological (SId, SIw) properties on the other hand. Growth traits were negatively correlated with density (μD and Pil from age 14 to age 51 months) and Cir59 was also negatively correlated with the three measurements of modulus of elasticity.

**Table 1:** Statistics for each wood quality related trait and growth: mean, standard deviation (SD) and coefficient of phenotypic variation (CPV).

Wood Properties	Trait	Trait definition (unit)	Mean	SD	CPV
Mechanical properties (M)	LGS	Mean Longitudinal Growth Strain (μm)	72	13	0.18
	E_p_	Primary value of Modulus of elasticity (GPa)	9	1	0.14
	E_30_	Mean Modulus of elasticity at 30 months (GPa)	11	1	0.12
	E_2i_	Modulus of elasticity after inflection point (GPa)	14	2	0.13
Technological properties (T)	SId	Splitting Index at 1 day	1.19	0.48	0.41
	SIw	Splitting Index at 1 week	0.96	0.49	0.51
	SIm	Splitting Index at 1 months	1.06	0.48	0.45
Physical properties (P)	Pil14	Pilodyn at 14 months (mm)	25	3	0.10
	Pil26	Pilodyn at 26 months (mm)	26	2	0.09
	Pil38	Pilodyn 38 months (mm)	25	3	0.11
	Pil51	Pilodyn 51 months (mm)	28	2	0.09
	Pil59	Pilodyn 59 months (mm)	27	3	0.11
	Pil59u	Pilodyn, under bark, 59 months (mm)	22	3	0.13
	μD	Mean microdensity (g.dm^-3^)	501	40	0.08
Chemical composition (C)	Text	Extractive content (%)	3.70	0.62	0.17
	Klass	Lignin content (wood %)	24.6	0.8	0.03
	G	G content (μmol.g^-1 ^lignin)	624	77	0.12
	S	S content (μmol.g^-1 ^lignin)	2504	405	0.16
	S/G	S/G ratio	4.03	0.53	0.13
	Poly	Polyphenols content (mg.g^-1^)	4551	1742	0.38
	Fla	Flavanols content (mg.g^-1^)	249	148	0.59
Anatomical properties (A)	FWL	weighted fibre length (mm)	1.28	0.05	0.04
	FW	Fibre width (μm)	33	1	0.03
	Coar	Fibre coarseness (μg.mm^-1^)	0.0966	0.0025	0.03
	Curl	Fibre curl index (%)	15.8	1.1	0.07
Growth (G)	Ht59	Total height at 59 month old (m)	24.2	2.4	0.10
	Cir59	Circumference (1.3 m) at 59 month old (cm)	54	9	0.16

**Table 2:** Matrix of significant Pearson correlation coefficients between studied traits (p < 1%)

		M				T			A				C							P							G
		
		LGS	E_p_	E_30_	E_2i_	SId	SIw	SIm	FWL	FW	Coar	Curl	Text	Klass	G	S	S/G	Poly	Fla	Pil14	Pil26	Pil38	Pil51	Pil59	Pil59u	μD	Ht59
M	E_p_	-																									
	E_30_	0.24	0.88																								
	E_2i_	-	0.46	0.70																							
T	SId	0.34	-	0.23	-																						
	SIw	0.41	-	0.29	-	0.74																					
	SIm	0.36	0.20	0.25	0.24	0.38	0.60																				
A	FWL	0.22	-	-	-	-	0.20	-																			
	FW	-	-	-0.28	-	-	-	-	-0.46																		
	Coar	-	-	-	-	-	-	-	0.73	-0.31																	
	Curl	-	-	0.21	-	-	-	-	-0.61	0.95	-0.50																
C	Text	-0.23	-	-	-	-	-	-	-0.21	0.24	-	0.23															
	Klass	-	-	-0.21	-	-	-	-	-	0.34	-	0.35	-														
	G	-	-	-	-	-	-	-	-	-	-	-	-	-													
	S	-	-	-	-	-	-0.19	-	-	-	-	-	-	-0.37	0.60												
	S/G	-	-	-0.20	-	-	-0.22	-	-	-	-	-	0.19	-0.29	-0.24	0.64											
	Poly	-	-	-	-	-	-	-	0.20	0.22	-	0.24	0.78	0.28	-	-	-										
	Fla	-	-	-	-	-	-	-	-	-	-	-	0.50	0.25	-	-	-	0.64									
P	Pil14	-	-0.55	-0.61	-0.49	-	-	-	-	-	-	-	-	-	-	-	-	-	-								
	Pil26	-	-0.47	-0.59	-0.51	-	-	-	-	-	-	-	-	-	-	-	-	-	-	0.61							
	Pil38	-	-0.32	-0.39	-0.44	-	-	-	-	-	-	-	-	-	-	-	-	-	-	0.50	0.65						
	Pil51	-	-0.40	-0.51	-0.52	-	-	-	-	-	-	-	-	-	-	-	-	-	-	0.48	0.66	0.65					
	Pil59	-	-0.20	-0.28	-0.22	-	-	-	-	-	-	-	-	-	-	-	-	-	-	0.32	0.48	0.51	0.59				
	Pil59u	-	-0.31	-0.34	-0.26	-	-	-	-	-	-	-	-	-	-	-	-	-	-	0.30	0.40	0.36	0.50	0.52			
	μD	0.23	0.43	0.58	0.51	-	-	0.30	-	-	-	-	-	-	-	-	-0.22	-	-	-0.43	-0.55	-0.42	-0.50	-0.35	-0.36		
G	Ht59	0.23	-	-	-	0.43	0.36	-	-	-	-	-	-	-	-	-	-	-	-	0.36	0.42	0.34	0.26	-	-	-	
	Cir59	-	-0.26	-0.28	-0.30	0.38	0.29	-	-	-	-	-	-	-	-	-	-	-	-	0.61	0.58	0.53	0.48	0.35	0.25	-0.26	0.83

Non-significant correlations are indicated by "-". Abbreviations are the same as in table 1.

### Genetic mapping

A total of 76 codominant markers were added to the earlier version of the maps constructed by Verhaegen and Plomion [33] and Gion et al. [34]. Additional file 1 - Table S1 summarizes the mapping information for these new markers including 3 SCARs, 13 SSRs 15 STSs, 54 ESTs, of which 27 were mapped in both parents, and 38 and 20 were mapped in *E. urophylla *and the *E. grandis *parents, respectively. In all, 116 and 110 framework markers were localized on the *E. urophylla *and *E. grandis *maps covering 1,383 and 1,216 cM, respectively (additional file 2 - Table S2). For both parents, 11 LGs were identified, corresponding to the 11 chromosomes of the *Eucalyptus *genome.

**Figure 2:**
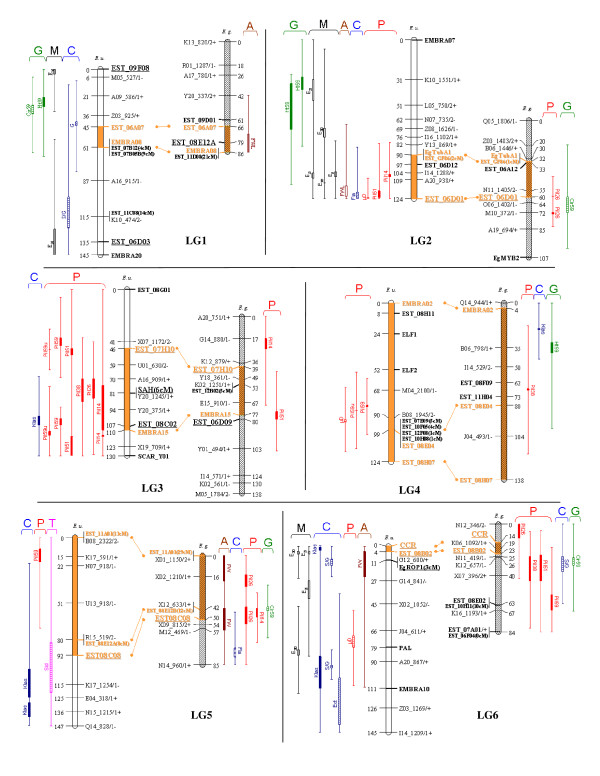
**Detection of QTLs for wood properties and growth related traits on the genetic maps of *E. urophylla *and *E. grandis *for the linkage groups 1 to 6**.

**Figure 3:**
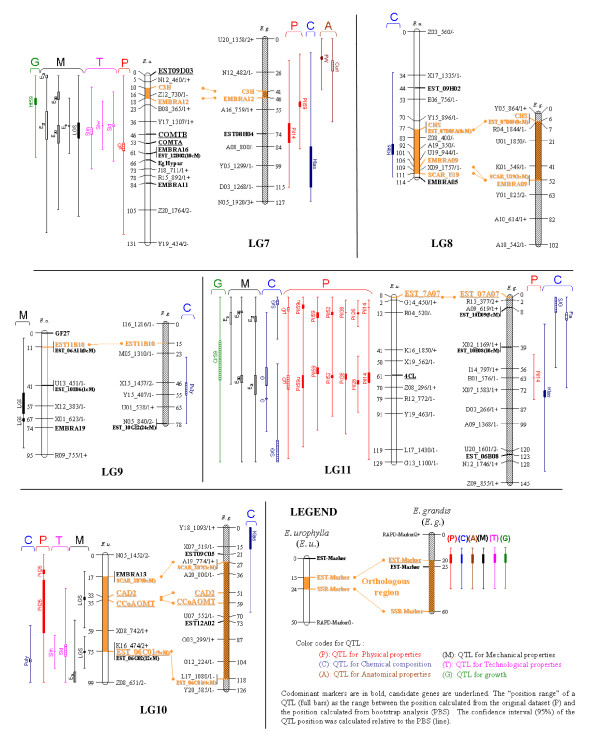
**Detection of QTLs for wood properties and growth related traits on the genetic maps of *E. urophylla *and *E. grandis *for the linkage groups 7 to 11**.

The codominant markers made it possible to confirm the assignment of orthologous LGs between parental maps previously inferred from intercross (low informative) dominant markers [33]. In all, 27 bridges were used to align the male and female LGs. Nine orthologous regions were identified covering 434 cM and 423 cM of the female (31%) and the male (35%) maps, respectively. This coverage percentage varied between LGs (see orange segments between orthologous markers in Figures 2 and 3).

### QTL analysis

#### Overview of the detected QTLs

QTLs were detected for the 27 traits analysed by multiple interval mapping in *E. urophylla *and/or *E. grandis *(Table 3 and Figures 2 and 3). In *E. urophylla*, 94 QTLs were detected, including 34 "single QTL" and 30 "two-linked QTLs". In *E. grandis*, 33 QTLs were detected, including 27 "single QTL" and 3 "two-linked QTLs".

**Table 3:** QTLs detected by multiple interval mapping in *E. urophylla *(*E.u*.) and *E. grandis *(*E.g*.) for i) mechanical properties; longitudinal growth strain (LGS) and modulus of elasticity (Ep E30, and E2i), ii) technological properties; splitting index (SId, SIs and SIm), iii) Physical properties (Pil, μD), iv) chemical properties; lignin content (Klas), monomeric composition (S, G, S/G), extractive contents (Ext), phenolic content (Poly) and flavanol content (Fla), and v) fibre properties; fibre weighted length (FWL) and fibre curl index (Curl), vi) growth, total height and circumference (1.3 m) at 59 months (Ht59, Cir59)

Species	Trait category	Trait	LG	n	LOD score	H_1_*vs*H_0_ H_2_*vs*H_0_	H_2_*vs*H_1_	QTL Model	P1 (cM)	P2 (cM)	d1	Δ1	d2	Δ2	PEV (%)	PEVtot (%)
*E.u*.	Mechanical properties (M)	LGS	7	198	3.5	0.0010	-	1	36	-	5.7	0.44	-	-	5.2	36.3
		LGS	9	198	4.7	0.0020	0.0080	2	63	69	24.5	1.88	-20.7	-1.59	19.5	
		LGS	10	198	6.9	< 10^-4^	< 10^-4^	2	33	75	4.8	0.37	-8.8	-0.68	11.7	
		E_p_	2	191	3.8	< 10^-4^	-	1	107	-	-0.73	-0.73	-	-	7.7	22.5
		E_p_	7	191	4.2	0.0010	-	1	40	-	0.78	0.78	-	-	8.9	
		E_p_	11	191	3.0	0.0010	-	1	12	-	0.63	0.63	-	-	5.8	
		E_30_	1	191	5.3	< 10^-4^	< 10^-4^	2	0	145	0.51	0.51	-0.50	-0.50	8.4	52.0
		E_30_	2	191	7.2	< 10^-4^	0.0010	2	76	112	0.25	0.25	-0.97	-0.97	12.5	
		E_30_	6	191	7.5	< 10^-4^	< 10^-4^	2	4	85	0.66	0.66	-0.63	-0.63	11.6	
		E_30_	7	191	9.3	< 10^-4^	< 10^-4^	2	13	39	0.53	0.53	0.47	0.47	11.6	
		E_30_	11	191	6.5	< 10^-4^	< 10^-4^	2	12	61	0.64	0.64	0.21	0.21	8.0	
		E_2i_	2	152	6.7	< 10^-4^	< 10^-4^	2	31	104	0.84	0.42	-1.27	-0.64	15.4	58.7
		E_2i_	6	152	5.1	< 10^-4^	< 10^-4^	2	4	27	1.26	0.63	-0.89	-0.45	8.1	
		E_2i_	7	152	9.6	< 10^-4^	< 10^-4^	2	0	28	0.93	0.47	0.69	0.35	14.5	
		E_2i_	10	152	3.4	0.0010	-	1	99	-	0.78	0.39	-	-	4.5	
		E_2i_	11	152	8.5	< 10^-4^	< 10^-4^	2	19	61	1.30	0.65	0.42	0.21	16.1	
	Technological properties (T)	SId	5	199	3.9	0.0030	0.0050	2	121	125	0.85	1.77	-0.92	-1.92	13.5	
		SId	7	199	4.4	< 10^-4^	-	1	38	-	0.28	0.58	-	-	8.6	
		SId	10	199	2.0	0.0090	-	1	79	-	-0.20	-0.42	-	-	4.1	26.0
		SIw	7	192	5.2	< 10^-4^	-	1	39	-	0.35	0.71	-	-	13.2	13.2
		SIm	7	190	4.0	< 10^-4^	-	1	46	-	0.28	0.58	-	-	8.6	
		SIm	10	190	4.0	< 10^-4^	-	1	75	-	-0.28	-0.58	-	-	8.5	17.1
	Physical properties (P)	Pil14	2	142	4.0	0.0010	-	1	105	-	1.36	0.45	-	-	6.7	
		Pil14	3	142	7.0	< 10^-4^	< 10^-4^	2	110	114	-4.37	-1.46	5.63	1.88	18.9	
		Pil14	11	142	11.0	< 10^-4^	< 10^-4^	2	8	59	-2.37	-0.79	-0.73	-0.24	25.4	51.1
		Pil26	3	140	3.4	< 10^-4^	-	1	70	-	1.09	0.55	-	-	5.3	
		Pil26	10	140	5.0	0.0010	0.0010	2	12	20	-4.33	-2.17	3.09	1.55	24.5	
		Pil26	11	140	12.5	< 10^-4^	< 10^-4^	2	12	64	-1.58	-0.79	-1.35	-0.68	23.4	53.2
		Pil38	3	199	2.8	0.0030	-	1	70	-	1.27	0.42	-	-	5.7	
		Pil38	11	199	10.0	< 10^-4^	0.0020	2	12	60	-1.91	-0.64	-1.08	-0.36	20.7	26.3
		Pil51	2	199	4.2	< 10^-4^	-	1	123	-	1.58	0.79	-	-	10.4	
		Pil51	3	199	5.6	< 10^-4^	0.0010	2	46	130	0.67	0.34	1.32	0.66	9.6	
		Pil51	11	199	9.7	< 10^-4^	< 10^-4^	2	12	61	-1.40	-0.70	-1.11	-0.56	16.4	36.4
		Pil59	3	198	8.6	< 10^-4^	< 10^-4^	2	44	101	1.05	0.35	1.13	0.38	12.8	
		Pil59	4	196	3.6	< 10^-4^	-	1	80	-	-1.31	-0.44	-	-	7.7	
		Pil59	5	198	2.7	0.0060	-	1	9	-	0.98	0.33	-	-	4.4	
		Pil59	11	198	9.0	< 10^-4^	< 10^-4^	2	12	55	-1.34	-0.45	-0.84	-0.28	14.5	42.5
		Pil59u	3	198	9.5	< 10^-4^	< 10^-4^	2	47	114	1.46	0.49	1.00	0.33	15.4	
		Pil59u	4	198	2.5	0.0050	-	1	84	-	-1.10	-0.37	-	-	5.3	
		Pil59u	11	198	6.9	< 10^-4^	< 10^-4^	2	6	61	-1.53	-0.51	-0.66	-0.22	13.8	34.5
		μD	2	199	4.9	< 10^-4^	-	1	124	-	-25	-0.63	-	-	9.9	39.5
		μD	4	197	3.4	< 10^-4^	-	1	93	-	18	0.45	-	-	5.4	
		μD	6	199	4.1	< 10^-4^	-	1	75	-	-21	-0.53	-	-	7.3	
		μD	7	199	4.3	< 10^-4^	-	1	53	-	20	0.50	-	-	6.3	
		μD	11	199	6.3	< 10^-4^	0.0010	2	8	61	21	0.53	10	0.25	10.5	
	Chemical composition (C)	Klas	3	189	4.2	0.0010	-	1	106	-	-0.43	-0.54	-	-	6.8	
		Klas	5	189	5.4	0.0010	0.0010	2	125	140	-0.78	-0.98	0.64	0.80	11.5	
		Klas	6	189	8.6	< 10^-4^	0.0060	2	1	111	-0.62	-0.78	0.15	0.19	14.7	
		Klas	8	189	3.1	0.0020	-	1	96	-	0.41	0.51	-	-	6	39
		S/G	1	189	3.2	0.0010	-	1	124	-	-0.24	-0.45	-	-	5	
		S/G	6	189	20.3	< 10^-4^	< 10^-4^	2	15	95	0.49	0.92	0.39	0.74	37	
		S/G	11	189	6.6	< 10^-4^	< 10^-4^	2	2	129	-0.26	-0.49	-0.21	-0.40	10	52
		Ext	6	188	2.7	0.0070	-	1	139	-	0.34	0.55	-	-	7.7	7.7
		G	1	189	3.7	< 10^-4^	-	1	41	-	44.3	0.58	-	-	8.3	
		G	11	189	5.4	< 10^-4^	0.0020	2	69	79	90.2	1.17	-84.5	-1.10	13.0	21.2
		Poly	10	183	2.5	0.0040	-	1	83	-	-1026	-0.59	-	-	8.7	8.7
		Fla	2	183	2.6	0.0010	-	1	124	-	-87	-0.59	-	-	8.7	8.7
	Anatomical properties (A)	FWL	2	177	3.5	0.0010	-	1	122	-	-0.04	-0.80	-	-	12.8	12.8
		FW	6	177	2.8	0.0070	-	1	4	-	-0.52	-0.52	-	-	7.1	7.1
	Growth (G)	Ht59	1	199	3.3	< 10^-4^	-	1	22	-	0.80	0.33	-	-	4.1	
		Ht59	2	199	21.2	0.0090	< 10^-4^	2	29	34	-5.78	-2.41	5.62	2.34	42.2	
		Ht59	7	199	4.0	< 10^-4^	-	1	18	-	0.90	0.38	-	-	4.7	51.0
		Cir59	1	198	5.1	< 10^-4^	-	1	28	-	5.8	0.64	-	-	11.9	19.5
		Cir59	11	198	2.7	0.0045	-	1	34	-	-4.7	-0.52	-	-	7.6	
*E.g*.	Physical properties (P)	Pil14	3	142	2.5	0.0100	-	1	17	-	-1.20	-0.40	-	-	5.3	
		Pil14	5	142	3.1	< 10^-4^	-	1	42	-	1.45	0.48	-	-	7.6	
		Pil14	7	142	2.5	0.0100	-	1	81	-	-1.45	-0.48	-	-	7.6	
		Pil14	11	142	4.4	< 10^-4^	-	1	63	-	1.70	0.57	-	-	10.5	31.1
		Pil26	2	140	5.8	< 10^-4^	< 10^-4^	2	59	72	2.92	1.46	-2.65	-1.33	18.6	
		Pil26	5	140	6.3	< 10^-4^	< 10^-4^	2	14	41	-0.64	-0.32	1.93	0.97	12.8	
		Pil26	6	140	3.7	< 10^-4^	-	1	0	-	1.27	0.64	-	-	7.4	38.8
		Pil38	4	199	3.1	0.0040	-	1	67	-	1.45	0.48	-	-	8.0	
		Pil38	6	199	2.8	0.0030	-	1	25	-	1.27	0.42	-	-	5.7	13.7
		Pil51	3	199	3.2	0.0010	-	1	80	-	1.36	0.68	-	-	7.6	
		Pil51	6	199	3.5	< 10^-4^	-	1	24	-	1.31	0.66	-	-	7.0	14.7
		Pil59	6	199	2.8	0.0020	-	1	67	-	1.14	0.38	-	-	5.9	
		Pil59	7	199	2.5	0.0040	-	1	53	-	-1.17	-0.39	-	-	6.3	12.2
	Chemical composition (C)	Klas	4	189	3.2	0.0010	-	1	20	-	-0.52	-0.65	-	-	9.5	
		Klas	7	189	3.3	0.0020	-	1	116	-	-0.43	-0.54	-	-	6.3	
		Klas	10	189	2.9	0.0030	-	1	0	-	-0.39	-0.49	-	-	5.3	
		Klas	11	189	3.1	0.0020	-	1	73	-	-0.40	-0.50	-	-	5.7	26.7
		S/G	6	189	2.9	< 10^-4^	-	1	37	-	0.28	0.53	-	-	7.2	
		S/G	11	189	2.9	0.0050	-	1	0	-	0.28	0.53	-	-	6.8	14.0
		Poly	9	183	2.5	0.0010	-	1	46	-	863	0.50	-	-	6.2	6.2
		Fla	5	183	3.3	< 10^-4^	-	1	79	-	90	0.61	-	-	9.6	
		Fla	11	183	4.4	< 10^-4^	-	1	12	-	100	0.68	-	-	11.8	21.4
	Anatomical properties (A)	FWL	1	166	3.1	0.0010	-	1	86	-	-0.03	-0.60	-	-	8.4	8.4
		FW	5	177	4.4	< 10^-4^	< 10^-4^	2	0	42	-0.47	-0.47	-0.59	-0.59	11.1	
		FW	7	178	4.0	< 10^-4^	-	1	14	-	-0.74	-0.74	-	-	13.9	25
		Curl	7	177	2.9	0.0020	-	1	17	-	-0.66	-0.60	-	-	11.1	11.1
	Growth (G)	Ht59	4	186	2.99	0.0010	-	1	31	-	1.34	0.56	-	-	7.9	7.9
		Cir59	2	198	2.60	0.0040	-	1	72	-	-3.7	-0.41	-	-	5.1	
		Cir59	5	198	2.80	0.0015	-	1	45	-	4.0	0.44	-	-	6.0	
		Cir59	6	198	3.42	< 10^-4^	-	1	26	-	4.2	0.47	-	-	6.8	17.9

QTLs location is given in cM from the top marker of the linkage group. Values of the maximum LOD-score and probability associated to the 1-QTL (H1 vs H0) or 2-QTL (H2 vs H0 and H2 vs H1) models are given for each QTL after 1,000 permutations. Difference between the two QTL allele effects (d1 and d2) also expressed in phenotypic standard deviations (Δ1 and Δ2). The percentage of phenotypic variation explained for each QTL (PEV) and the total PEV are also provided.

Among the 94 QTLs detected at the 10% genome wide level, most (87%) were still significant at the 5% genome wide level. As shown in additional file 3 - Figure S1, 72% of the WP-QTLs were detected into five LGs (LG2, LG3, LG6, LG7 and LG11). For growth, 50% of the QTLs were detected on LG1 and LG2.

While 62% of the QTLs had a rather limited effect (percent of phenotypic variance, PEV < 10%), 38% displayed an effect ≥ 10%. The strong QTL effect (PEV > 10%) represented 47% of the QTLs detected in *E. urophylla *but only 23% of those detected in the *E. grandis*. The PEV explained by each QTL varied from 4.1% to 42.2% for *E. urophylla *and from 5.3% to 18.6% for *E. grandis*. Maximum total PEV amounted to 58.7% in *E. urophylla *for E_2i _and 38.8% in *E. grandis *for Pil26. However, given the size of the sample used in this study (between 140 to 199 trees phenotyped), it should be noted that the PEVs are probably biased upwards [64]. This is clearly illustrated in *E. urophylla *for Pil14 (142 trees phenotyped), Pil26 (140 trees) and E_2_i (152 trees), for which the total PEV were the highest.

#### QTLs detected for each WP category

##### Mechanical and technological properties

In *E. urophylla*, 34 QTLs (5 for LGS, 4 for SId, 1 for SIw, 2 for SIm, 3 for E_p_, 10 for E_30 _and 9 for E_2i_) were detected. The total PEV varied between 13.2% and 58.7%. Three major QTL clusters were identified on LG7, LG10 and LG11. In *E. grandis *no QTL was detected.

##### Physical properties

In *E. urophylla*, 35 wood density QTLs were detected with a total PEV varying from 26.3% for Pil38 (3 QTLs) to 53.2% for Pil26 (5 QTLs). In *E. grandis *15 QTLs were detected with a total PEV varying from 13.7% for Pil38 (2 QTLs) to 38.8% for Pil26 (5 QTLs). Three major QTL clusters were identified at all the measurements dates on LG3 and LG11 for *E. urophylla *and on LG6 for *E. grandis*, reflecting the high phenotypic correlations between these traits. The linkage phase between QTLs and flanking markers was the same for the two clusters, only one change in the linkage phase was observed for Pil14 in LG3.

##### Chemical composition

In *E. urophylla*, with the exception of S content, QTLs were detected for all the traits (17 QTLs in total). Total PEV varied from 7.7% for Ext (1 QTL) to 52% for S/G ratio (5 QTLs). In *E. grandis *far fewer QTLs were detected: 4 QTLs for lignin content (Klas), 2 for S/G ratio, 1 for Poly and 2 for Fla. Total PEV varied from 6.2% for Poly to 26.7% for Klas. Two clusters in LG6 and LG11 were identified on both parental maps. Interestingly, the 10 QTLs detected for lignin content were localized on 8 different chromosomes. S/G ratio QTLs shared orthologous genomic positions on LG6 and LG11 on both maps.

##### Anatomical properties

In *E. urophylla*, 1 QTL was detected for FWL (12.8%, LG2). In *E. grandis*, 1 QTL for FWL (8.4%, LG1) and 1 QTL for Curl (11.1%, LG7) were detected. No QTL was significant for Coar.

##### Growth

In *E. urophylla*, 4 QTLs were detected for growth on LG1, LG7 and LG11 explaining from 4.1% to 11.9% of phenotypic variation. Two-linked QTLs were also detected on LG2 accounting for 42.2% of phenotypic variance. In *E. grandis*, only 4 QTLs were detected for growth (7.9% for Ht59 on LG4 and 5.1 to 6.8% for Cir59 on LG2, LG5 and LG6).

#### Co-locations between QTLs

Two types of QTL co-locations were observed. First, "expected" co-locations consistent with significant phenotypic correlations found between traits. A total of 97 such "expected" co-locations between WP QTLs were observed in *E. urophylla *and 3 in *E. grandis*, 44% being wood density QTLs. Indeed, wood density was measured at different ages and these traits presented relatively high phenotypic correlations (Pearson coefficient ranging from 0.32 to 0.66). Co-located QTLs were mainly distributed in LG 3 and LG11 in *E. urophylla *and LG6 in *E. grandis*. Mechanical and technological properties (LGS, SI and E traits) also showed high correlations (0.34 - 0.74) and co-located QTLs in *E. urophylla *(LG7 and LG10). The same result was observed on LG11 with QTL co-locations for Pil and E, which were negatively correlated. However, it should be noted that significant correlation did not always result in co-located QTLs. This was the case for Ht59 and Pil in both parents, despite significant correlations (0.26-0.42) and between Ht59 and mechanical properties (0.23) or technological properties (0.36-0.43).

More surprisingly, "unexpected" co-locations were observed between uncorrelated traits (23 in *E. urophylla *and 5 in *E. grandis*), in *E. urophylla *on LG2 between wood density (Pil51 and μD), chemical composition (Fla) and anatomical characteristics (FWL), on LG5 between technological (SId) and chemical (Klass) properties, on LG6 between chemical (S/G) and wood density (Pil38 and Pil51), and on LG11 between the cluster of WD QTLs and chemical properties (S/G and G content); in *E. grandis *on LG5 between density (Pil14, Pil26) and FW, on LG6 between WD (Pil38 and Pil51) and S/G ratio.

Overall, multiple co-locations were much more frequent for pairs of correlated traits (31 pairs showing at least 2 co-locations vs. 27 pairs with one co-location) compared to non-correlated traits (1 pair with at least 2 co-locations vs. 26 pairs with one co-location).

Finally, the probability that these co-locations were observed by chance is shown in Additional file 4 - Table S3 for the different pairs of traits. On average, the mean interval length as defined in the Material and Methods section was 8.5 cM for *E. urophylla *and 9.6 cM for *E. grandis*. The length of the female map was 1,383 cM, and the number of intervals that could be compared was 163. The number of intervals on the male map was 127 (1,216 cM). For 79% of the co-locations observed for traits harbouring significant correlations and 29% of the co-locations detected between uncorrelated traits, probabilities were lower than 5% suggesting that these observed co-locations are not due to chance alone.

#### Co-locations between specific trait-QTLs and candidate genes (CGs)

The probability that co-locations of QTLs and CGs occurred by chance was estimated considering all traits, all genes and all LGs for each parent. In *E. urophylla*, considering 94 QTLs and 40 CGs, the probability of 36 co-locations (involving 18 CGs) was < 10^-4^, indicating a non random association. For *E. grandis*, considering 33 QTLs and 34 CGs, the probability of 9 co-locations (involving 7 CGs) was 0.1791, meaning these coincidences could not be considered as a non-random event.

In *E. urophylla*, out of the 18 CGs displaying such a coincidence (Table 4), five corresponded to known lignin biosynthetic genes (CCR, COMT, CAD2, CCoAOMT, 4 CL), two genes had a possible indirect effect on lignification (EgROP1 and SAH), 3 presented similarities with known function proteins (subtilisin-like proteinase, xyloglucan endo glycosylase, 60 S Ribosomal protein L7), while two corresponded to unknown proteins and 6 had no match in public databases. Co-locations of lignification genes were observed for different categories of traits indicating a possible genetic link between such traits and the lignification pathway: CCR co-located with one of the two QTLs detected for Klas on *E. urophylla *LG6. In the same linkage group, EgROP1 co-located with a QTL for S/G. COMT (LG7) and CAD2 (LG10) co-located with two QTLs for LGS explaining 5.7% and 11.7% of the phenotypic variance respectively. Three lignification genes (CAD2, CCoAOMT and 4 CL) mapped on LG10 and LG11 and co-located with QTLs for Pil at different ages. Finally a co-location was found for LG11 between a putative xyloglucan endotransglycosylase (EST07A07) and a QTL for the S/G ratio not only in *E. urophylla *but also in *E. grandis*. Considering, the 7 structural genes of the lignification pathway mapped in *E. urophylla *(COMT, CCoAOMT, 4 CL, PAL C3H, CCR, CAD) and QTLs for lignin related traits (5 QTLs for S/G, 6 QTLs for Klas, 3 QTL for G, representing 12 independent QTL regions). The prior probability to obtain the two co-locations by chance alone was barely significant (p = 0.0759).

**Table 4:** Co-locations between candidate genes and QTLs in *E. urophylla *and *E. grandis*

LG	Marker ID	Fonction	*E. urophylla*		*E. grandis *(NS)	
			Trait-QTL	PEV (%)	Trait-QTL	PEV (%)
1	EST09F08	unknown	E_30_	8.4^a^		
	EST06D03	60 S Ribosomal protein L7	E_30_	8.4^a^		
	EST08E02	unknown			FWL	8.4
2	EST06D01	unknown	Pil51; FWL; Fla; μD	10.4; 12.8; 8.7; 9.9	Pil26	18.6
3	EST08C02	No hit	Pil14, Pil59, Klass	6.7^b^; 12.8; 6.8		
	EST07H10	No hit	Pil51	9.6		
	EST06D09	unknown			Pil51	7.6
	SAH	adenosylhomocysteinase	Pil14, Pil26, Pil38	6.7^b^; 5.3; 5.7		
5	EST08C08	No hit	SId	13.5	Pil14, Pil26, FW	7.6; 12.8; 11.1
6	CCR	lignification	Klass	14.7		
	EST08E02	Phytochelatin synthetase			Pil59	5.9
	EST08B02	No hit	FW, E_30_, E_2i_	7.1^c^; 11.6; 8.1		
	EgROP1	Regulation	FW, S/G	7.1^c^; 37.0		
7	COMT	lignification	LGS, E_30_, E_2i_	5.2; 11.6; 14.5^d^		
	EST08H04	unknown			Pil14	7.6
	EST09D03	subtilisin-like proteinase	E_2i_	14.5^d^		
10	CAD2	lignification	LGS, Pil26	11.7; 24.5^e^		
	CCoAOMT	lignification	Pil26	24.5^e^		
	EST06C01	no hit	SId	4.1		
11	4CL	lignification	Pil14, Pil38, Pil51, μD, G, E_30_, E_2i_	25.4; 20.7; 16.4; 10.5; 13.0; 8.0; 4.5		
	EST07A07	Xyloglucan endotransglycosylase	S/G	10.0	S/G	6.8

The PEV values are given for the corresponding QTLs.

a, b, c, d and e indicate that the same QTL co-localized with different candidate genes.

## Discussion

### Genetic architecture of wood properties-QTLs

**Figure 4:**
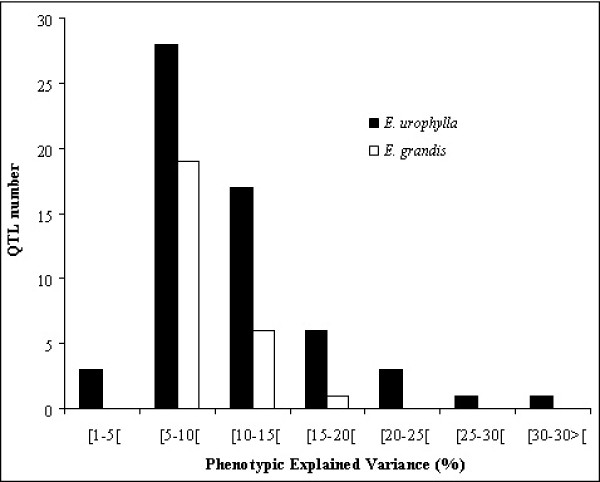
**Distribution of effect classes for all wood property QTLs detected in *E. urophylla *and *E. grandis***.

A total of 117 QTLs were detected for 26 WP-traits, *i.e*. an average of 4.5 QTLs per trait, which is consistent with the hypothesis that wood properties are under polygenic control. The difference in terms of detected QTLs in *E. grandis *and *E. urophylla *could be due to differences in level of heterozygosity between the two parents, as illustrated by the mapped codominant markers, heterozygosity being higher in *E. urophylla *(76%) than in *E. grandis *(53%). The mean number of QTLs varied according to the category of WP: Physical properties (Pyl and μD) presented the highest number of QTLs (an average of 7 QTLs per trait) followed by mechanical (5.4 QTLs), chemical (3.7 QTLs), technological (2.3 QTLs) and anatomical (1.7 QTLs) properties. While most QTLs accounted for a rather low fraction of the phenotypic variation of WPs, 12 genomics regions (2 linked QTLs in each region) presented a PEV greater than 15%. The L-shaped QTL distribution (Figure 4) obtained in the present study is consistent with other QTL studies involving similar sample size for low to medium heritability traits [65].

Thamarus et al. [24] published the first QTL study in *Eucalyptus *for a series of wood properties. These authors focused on three classes of WPs, namely chemical, density and fibre properties. They detected a total of 13 regions with an effect on density, chemical properties (cellulose content) and pulp and paper properties (pulp yield, fibre length and microfibrille angle) and some of them were found in two pedigrees of *E. globulus*, indicating a stable effect of these genomic regions. The detected effect explained from 3.2 to 15.75% of the phenotypic variance. In more recent investigations, Freeman et al. [26] detected 11 QTLs in *E. globulus *explaining between 5.6 to 12.3% of the phenotypic variation of WPs and Thumma et al. [27] detected 36 QTLs in *E. nitens *with minor effects (< 10% of the PEV). Even if large effect QTLs are biased upward because of the relative small population sizes used in most QTL studies [64], these results are encouraging for the characterization of the nature of QTLs with a major effect on wood properties using a map-based cloning approach. As more QTL studies accumulate, comparative QTL mapping will help to validate the presence of stable QTLs across the genus. We believe that such QTLs will be good targets for map-based cloning for a more fundamental aspect.

### Power of QTL detection

For a given sample size of a given genetic background and for a given QTL detection method, the power of QTL detection depends on trait complexity, the extent of phenotypic variance of the trait, and the genetic parameters of the trait (heritability and the proportion of additive vs. non-additive variance).

Regarding trait complexity, chemical properties are likely to be less complex than integrated traits such as wood density or growth. Indeed, chemical properties often involve a single biosynthesis pathway consequently the number of QTLs is expected to be lower than for traits that are affected by many different physiological processes. The results of the present study agree with this hypothesis, the average number of QTLs being much higher for physical properties (7) than for chemical properties (3.7). Regarding the relationships between phenotypic variance and the number of QTLs, the following observations were made: i) the five traits with the highest CPV (> 0.38) presented fewer QTLs (2.4 on average) than the traits with a lower CPV (< 0.18, 4.9 QTLs on average). We attributed such high CPV for all technological (SId, SIw, SIm) and the two chemical (Fla and Poly) traits to a combined effect of intrinsic variability and environmental variations, including inaccuracy in trait measurement, ii) when considering only traits with a CPV < 0.18, we did not detect any relationship between the number of QTLs and the level of phenotypic variation. This result also held true when all the traits were taken into consideration (additional file 5 - Figure S2). The same trends were reported by Thumma et al. [27], Thamarus et al. [24], and Freeman et al. [26] for a similar set of WPs in *E. nitens *and *E. globulus*. Therefore the extent of phenotypic variation apparently did not strongly influence the number of QTLs detected in our study.

In a full-sib progeny obtained from the control cross of highly heterozygous parents, analysis of marker-trait associations using dominant or co-dominant markers in a test-cross configuration (1:1 segregation), enables detection of QTLs for each parent of the cross and the null hypothesis tests an allelic substitution averaged over the alleles inherited from the other parent [20], i.e. an effect combining additivity and dominance. In such an experimental system, the power of QTL detection will not only depend on the level of narrow sense heritability of the trait [66] but will also be strongly affected by the extent of dominance. In our study, growth and chemical related traits presented a similar number of QTLs despite different levels of narrow sense heritabilities (additional file 6 - Table S4) and a different extent of additive variance [15]. We conclude that it remains impossible to predict the number of QTLs in a given context on the basis of trait variability and prior knowledge on genetic parameters.

### Co-locations between QTLs for correlated WPs suggests pleiotropic effect rather than physical linkage

We tested whether QTLs collocate assuming the null hypothesis that they are randomly and independently distributed. Overall, we rejected the null hypothesis in 52% of the case but this ratio was much higher for correlated traits (65%) than for uncorrelated traits (25%), suggesting as expected that correlated traits shared more often spatial position of QTLs than in the case of uncorrelated traits.

Two classical hypotheses can be proposed to explain significant co-locating QTLs: Pleiotropy (i.e. several traits controlled by a single gene) *vs*. physical linkage (i.e. cluster of genes controlling different traits). In respect to correlated traits, the fact that 79 significant co-locations (most occurring several times in different regions) with similar allelic effects between different WPs were identified, suggests a common molecular basis for the genetic control of these correlated traits. Indeed, if a single co-location of 2 QTLs could be due to chance alone, the observation of multiple co-locations could be considered as a good indication of a common genetic control of the different WPs, reflecting the effects of pleiotropic genes rather than the presence of several linked genes. This was particularly clear for wood density measured at different maturation stages (from age 14 to 59 months), showing multiple occurrences of Pil QTLs. This result also suggests that the genetic control of wood density is quite stable during ontogenic development. The presence of stable QTLs for wood density is further supported by the presence of co-locating QTLs for μD (an integrated measurement of wood density across the whole tree ring) in the three distinct chromosomal regions harbouring QTLs for Pil (i.e. in LG2, 4 and 11). Co-located QTLs for non-correlated traits were less frequent and usually involved a single chromosomal region. In this case, "linked genes" is a more likely hypothesis and such coincidences simply result from the strong linkage disequilibrium (LD) between distant loci in the mapping pedigree. In the coming years, fine QTL mapping [67] combined with knowledge of the *Eucalyptus *genome sequence [http://web.up.ac.za/eucagen] should improve our understanding of the nature of the detected QTLs and disentangle pleiotropy versus physical linkage.

### The presence of QTLs in orthologous regions validate the presence of generic genomic regions for WPs

Homologous LGs between the male (*E. grandis*) and female (*E. urophylla*) maps were identified using codominant markers. A total of nine orthologous regions (representing 31% and 35% of the total length of *E. urophylla *and *E. grandis *genetic maps, respectively) were identified based on multiple parallel linkages of orthologous markers, making it possible to identify similar genomic regions controlling WPs. Hot spots of QTLs found in both species were thus identified on LG5, LG6, LG7 and LG11 (Figures 2 and 3). Thumma et al. [27] also reported co-location of QTLs for fibre properties in *E. nitens *and for wood density in *E. globulus *for two orthologous QTL regions. In this paper, we were able to identify several orthologous regions with the same trait-QTLs between *E. urophylla *and *E. grandis *(e.g. for Pil51 in LG3 and for S/G ratio in LG6 and LG11) which is a good indication of the reliability of the detected QTLs.

Comparing our study with that of Thumma et al. [27], two orthologous regions between *E. urophylla*, *E. grandis*, *E. nitens *and *E. globulus *were identified harbouring WP-QTLs: i) wood density QTLs in the region surrounding microsatellite marker EMBRA15 mapped on LG3 in *E. urophylla *and *E. grandis*, and on LG8 in *E. nitens *and *E. globulus*, and ii) chemical and MFA QTLs around the CCR gene mapped on LG6 in *E. urophylla *and *E. grandis *and LG10 in *E. nitens *and *E. globulus*. A table of correspondence between LGs of this study and that of Brondani et al. [50] based on SSR markers is available in additional file 7 - Table S5. The clustering of WP-QTLs in orthologous genomic regions in different species indicates that these regions could be of general interest for the *Eucalyptus *genus and should be considered as important targets for QTL dissection. The presence of stable regions for WPs across species is also coherent with results of quantitative genetic analyses which generally indicate that WPs present higher narrow sense heritability than growth related traits (reviewed by Raymond [4]). Even if heritability varies between WPs and depends to a great extent on the genetic context and environmental conditions of the trials, most results (reviewed in Additional file 6 - Table S4 for *Eucalyptus *species) agree with a medium to strong genetic control for WPs.

### Decoding the genes underlying QTLs

In our study, we detected co-locations between expressional (e.g. transcripts over-expressed in differentiating xylem as compared to other tissues, [68]) or functional (i.e. genes of known function) candidate genes and QTLs for physical and chemical properties in *E. urophylla *and *E. grandis*. Although we are aware that many genes are possibly involved, because of the large QTL confidence intervals (several cM), we believe that some co-locations would merit further investigations because they are supported by additional sources of evidence from reverse and forward genetics experiments or functional genomics (transcriptomics, proteomics) studies.

The most notable co-location was found in *E. urophylla *(LG6) of a gene encoding a structural gene of the lignification pathway (CCR) and a QTL for lignin content (Klas). Associations between CCR and WPs have been already reported in *Eucalyptus*. Thamarus et al. [24] and Thumma et al. [27] reported co-locations of CCR and QTLs for pulp yield and cellulose content (in *E. globulus*) and MFA (in *E. nitens*) respectively. Using an association genetics approach, Thumma et al. [69] and Mandrou et al. [A candidate gene for lignin composition in *Eucalyptus*, submitted] detected effects of nucleotide variability in CCR on MFA (in *E. nitens) *and S/G ratio (in *E. urophylla*), respectively. The existence of a single CCR gene in *Eucalyptus *[70,71] reinforces the hypothesis of a single orthologous region surrounding CCR with an effect on several WPs across different genetic backgrounds. The association between CCR, or surrounding gene polymorphisms in LD with CCR, and a series of uncorrelated WPs in different species, suggests the presence of a gene cluster controlling different wood properties in this chromosomal region. As soon as the genome sequence of several *Eucalyptus *species is available, regional association mapping with marker saturation around the CCR locus will enable more precise characterization of the polymorphisms involved in variations of wood property. Other lignification genes (4 CL, COMT, CAD2 and CCoAOMT) co-located with mechanical (LGS, E) and technological (SI) wood properties in *E. urophylla *(LG7, LG10, LG11), which is coherent with the high correlations observed between wood chemistry (especially lignin content and composition) and ultrastructural properties (LGS, MFA) [3,72]. Co-location of lignification genes (4 CL, C 3 H, C4H and CCoAOMT) and WP (wood specific gravity) was also observed by Brown *et al*. [73] in *Pinus taeda*.

Another noteworthy co-location concerns EgROP1 (a member of the plant ROP family of Rho-like GTPases) and a QTL for S/G ratio in *E. urophylla *(LG6). Using a transgenesis approach in *Arabidopsis thaliana*, Fourcart et al. [32] found that plants overexpressing this gene exhibited larger vessel-like cells containing G units and hypothesized that this signaling protein might affect lignin composition by influencing the vessel to fibre ratio. Using an association mapping approach, Mandrou et al. [A candidate gene for lignin composition in *Eucalyptus*, submitted] also found that a SNP in this gene was associated with the S/G ratio, providing an additional line of evidence of the importance of this regulatory gene in controlling lignin quality.

## Conclusion

This report constitutes the most extensive contribution (in terms of number of traits analyzed) to the understanding of the genetic architecture of wood properties in the most planted tree species worldwide, and opens great perspectives to decipher the nature of the underlying genes, the eucalyptus genome being now available (http://www.phytozome.net/eucalyptus.php). Several genomic regions with major effects were detected for all the studied traits. The co-location between QTLs for different WP categories, suggests the existence of generic genomic regions corresponding to clusters of genes with independent effects on different WPs or genes with pleiotropic effects. Some co-locations between known function genes (e.g. CCR) and WP QTLs (e.g. lignin content) agrees with previous results using forward or reverse genetic approaches, and contribute to increase our knowledge about the nature of quantitative trait loci controlling wood quality.

## Authors' contributions

JMG, AC, SD, CB, FB: Genotyping and genetic mapping analysis; JMG, FP, JPC, HB, PR, NO: wood properties phenotyping; JMG, VC: primer design for EST markers; JMG: QTL analysis; JMG, CP: manuscript preparation; JMG, DV, JGP, PV, CP: project design, funding and overall supervision. All authors have read and approved the final manuscript

## Supplementary Material

Additional file 1 **Table S1**: Linkage group location of codominant markers (EST, SSR and STS) on *E. urophylla *and *E. grandis *linkage maps based on their segregation in the interspecific F1 progeny. Click here for file

Additional file 2 **Table S2**: Characteristics of *E. urophylla *and *E. grandis *genetic maps. The total length (in cM) of each linkage group (LG) was obtained using the Kosambi function. The total number of framework markers (Fr markers) and codominant markers (Co markers) are provided. Click here for file

Additional file 3 **Figure S1**: Distribution of WP and growth QTLs on the linkage groups of *E. urophylla *and *E. grandis*. Click here for file

Additional file 4 **Table S3**: Probability of a random co-location of QTL pairs in *E. urophylla *(*E.u*) and *E. grandis *(*E.g*). Click here for file

Additional file 5 **Figure S2**: Number of detected QTLs as a function of the coefficient of phenotypic variation (CPV) for all the traits. Click here for file

Additional file 6 **Table S4**: Literature review of estimated heritability for wood properties and growth in *Eucalyptus *species. Click here for file

Additional file 7 **Table S5**: Correspondences between linkage groups (LGs) of this study and that of Brondani et al. [50] based on SSR markers. Click here for file
