# Supplementary material for: Comprehensive genetic dissection of wood properties in a widely-grown tropical tree: Eucalyptus
Source: BMC Genomics. 2011 Jun 8;12:301. doi: 10.1186/1471-2164-12-301 (PMC3130712; doi:10.1186/1471-2164-12-301)
Supplement: Additional file 1 — Table S1: Linkage group location of codominant markers (EST, SSR and STS) on E. urophylla and E. grandis linkage maps based on their segregation in the interspecific F1 progeny. [file 1471-2164-12-301-S1.PDF]

Supplementary table S1 : Linkage group location of codominant markers (ESTs, SSRs and STSs) on the *E. urophylla* and *E. grandis* linkage maps based on their segregation in the interspecific F1 progeny. The significance of segregation distorsions are indicated in the $\chi\iota^2$ column (* 5%, ** 1% and *** 0.01%).

| Marker ID | Marker type | Homology | Function | GenBank Acc. | primer pairs | Number of offsprings | Segregation ratio | $\chi_i^2$ | df | Parental Maps | *LG* |
|---|---|---|---|---|---|---|---|---|---|---|---|
| Myb2 | STS | MYB transcription factor | Cell Wall | AJ576023 | Goicoechea *et al.* 2005 | 191 | 1.1.1.1 | 1.5 | 3 | *E.u. E.g.* | LG2 |
| CCR | STS | cinnamoyl-CoA reductase gene | Cell Wall | X97433 | Gion *et al.* 2000 | 164 | 1.1.1.1 | 0.12 | 3 | *E.u. E.g.* | LG6 |
| C3H | STS | p-coumarate 3-hydroxylase | Cell Wall | AJ438348 | F:ACGACCTCAGCGAAGATACC<br>R:GATGGCCCATACATTCACG | 94 | 1.1.1.1 | 0.21 | 3 | *E.u. E.g.* | LG7 |
| CAD2 | STS | cinnamyl alcohol dehydrogenase | Cell Wall | X75480 | Gion *et al.* 2000 | 170 | 1.1.1.1 | 3.64 | 3 | *E.u. E.g.* | LG10 |
| CCoAOMT | STS | caffeoyl-CoA O-methyltransferase | Cell Wall | Y12228 | Gion *et al.* 2000 | 176 | 1.1.1.1 | 3.22 | 3 | *E.u. E.g.* | LG10 |
| 12H09 | EST | pectinacetylesterase, putative | Cell Wall | AJ627868 | F:GACTGTCCCTACCCTTGC<br>R:GCTCTCTTATCCCCTTTG | 90 | 1.1.1.1 | 1.66 | 3 | *E.u. E.g.* | - |
| 12B02 | EST | glycine-rich protein | Cell Wall | AJ627840 | F:CTGATGTTTATCATTATCTGCCTCC<br>R:GCAGTTATCCATATTACAGCCCAG | 89 | 1.1 | 0.55 | 1 | *E.u.* | LG7 |
| GF27 | EST | xyloglucan endo-1, 4-beta-D-glucanase | Cell Wall | n.a. | F:TTATGTGGATGAAGTGCCC<br>R:AACTTGAGCCTCCCAGTGT | 168 | 1.1 | 0.21 | 1 | *E.u.* | LG9 |
| 06A11 | EST | xyloglucan endo-1,4-beta-D-glucanase | Cell Wall | AJ627651 | F:GATAGTGAAAAGTGAGGCAATGG<br>R:ACCGCAGAGTGTCGTAGCAGTAG | 85 | 1.1 | 0.95 | 1 | *E.u.* | LG9 |
| 08E04 | EST | Cellulase | Cell Wall | AJ627734 | F:GCCTGCACTTACAATACCGC<br>R:GATTACTACACGGGCTTCACAA | 172 | 1.1.1.1 | 10.56* | 3 | *E.u. E.g.* | LG4 |
| 07A07 | EST | xyloglucan endotransglycosylase XET1 | Cell Wall | AJ627694 | F:GCAAAGGCAATAGGGAGCAGC<br>R:AGGAAGAAGGGCAAGTGGAGG | 86 | 1.1.1.1 | 6 | 3 | *E.u. E.g.* | LG11 |
| PAL1 | STS | phenylalanine ammonia lyase | Cell Wall | D30656,D83075,l11747,l33677,<br>U39792,U43338,X52953,X16099<br>,X58180,X78269 | Gion *et al.* 2000 | 85 | 1.1 | 1.99 | 1 | *E.u.* | LG6 |
| COMTB | STS | caffeic acid O-methyltransferase | Cell Wall | X74814 | Gion *et al.* 2000 | 180 | 1.1 | 0.36 | 1 | *E.u.* | LG7 |
| COMTA | STS | caffeic acid O-methyltransferase | Cell Wall | X74814 | Gion *et al.* 2000 | 180 | 1.1 | 0.02 | 1 | *E.u.* | LG7 |
| 4CL | STS | 4-coumarate:CoA ligase | Cell Wall | AJ244010 | Gion *et al.* 2000 | 144 | 1.1 | 1.00 | 1 | *E.u.* | LG11 |
| 09A06 | EST | Xylanase | Cell Wall | AJ627746 | F:GGGAACGAGGACATACC<br>R:TGGAGCCACAGACACTT | 84 | 1.1 | 1.7 | 1 | *E.g.* | LG2 |
| 12B02 | EST | glycine-rich protein | Cell Wall | AJ627840 | F:CTGATGTTTATCATTATCTGCCTCC<br>R:GCAGTTATCCATATTACAGCCCAG | 89 | 1.1 | 2.53 | 1 | *E.g.* | LG3 |
| 10G12 | EST | Cellulose synthase CesA-1 | Cell Wall | AJ627788 | F:GAACAGGACTCCGACCATTG<br>R:CGCAAAACTGCAAAAACAGC | 93 | 1.1 | 25.82*** | 1 | *E.g.* | LG9 |
| 11D08 | EST | putative glucosyltransferase | Metabolism | AJ627813 | F:CGGATGCTCGTGAGGATTTC<br>R:ACGGGTTCGTGAGCGGGGAC | 84 | 1.1 | 0.43 | 1 | *E.g.* | LG1 |
| 10E06 | EST | beta-Amyrin Synthase | secondary metabolism | AJ627778 | F:TACACTATGCCGCTTACAGG<br>R:TTGGATCCTCAGGTTCTTG | 90 | 1.1 | 0.04 | 1 | *E.u.* | LG9 |
| 10C04 | EST | putative pseudouridine synthase | Nucleotide metabolism | AJ627770 | F:CGAAGCGAGGACTGAAGAAG<br>R:CGACCGATGATTTGATAGCC | 174 | 1.1 | 1.47 | 1 | *E.u.* | LG3 |
| 11H04 | EST | dTDP-glucose 4,6-dehydratase | Nucleotide metabolism | AJ627834 | F:ATGCGTTATTACACAAGG<br>R:GATTTCAAGTGGGTCAAC | 85 | 1.1 | 0.11 | 1 | *E.g.* | LG4 |
| 07A01 | EST | cobalamine-independent methionine synthase | Amino acid metabolism | AJ627690 | F:GTGGAATGTTCATCTCGC<br>R:GTGAAATATGGTGCTGGC | 78 | 1.1 | 0.21 | 1 | *E.g.* | LG6 |
| 10H08 | EST | probable ABC transporter | ABC transporters | AJ627795 | F:ACGTGCTATGGTGAAGGATC<br>R:TGGGAAAGGAGGACACAAC | 93 | 1.1 | 0.87 | 1 | *E.u.* | LG4 |
| 12A02 | EST | putative aquaporin PIP2-2 | Other transport facilitators | AJ627837 | F:TACAACAACGGCAAAGTG<br>R:TCACCATCCATCTCCATC | 88 | 1.1 | 0.18 | 1 | *E.g.* | LG10 |
| 09D01 | EST | transformer serine/arginine-rich ribonucleoprotein, putative | mRNA Transcription | AJ627752 | F:ATCGAGGAGGCATACGACC<br>R:CGGCGGAGGCAGAATAGAC | 171 | 1.1 | 1.32 | 1 | *E.g.* | LG1 |
| 00B00 | EST | COP9 complex subunit 2 | Other | n.a. | unpublished | 92 | 1.1.1.1 | 1.14 | 3 | *E.u. E.g.* | LG5 |
| 06D12 | EST | protein serine/threonine kinase | Kinase | AJ627669 | F:TTGCAGTTCAGCCCCCTCC<br>R:ATGCGAAGCGACTCCCCTC | 81 | 1.1 | 0.6 | 1 | *E.u.* | LG2 |
| 10F05 | EST | leucine-rich receptor-like protein kinase - like protein | Kinase | AJ627784 | F:AGGAGATGATCCAGATGCTG<br>R:CCTATACCAATCCCAACCC | 92 | 1.1 | 0.7 | 1 | *E.u.* | LG4 |
| 07E09 | EST | RING zinc finger | Other | AJ627710 | F:ATTGGGTGTGCTGCGTGT<br>R:TCTCAGGTTTGGGGTAGGAA | 78 | 1.1 | 3.28 | 1 | *E.u.* | LG4 |
| EgROP1 | EST | Rac-like small GTPase | GTP binding protein | EF392836 | F:GATCCGCATTTTCCCCATCGC<br>R:CAAGCCACGCCAATTCAACC | 93 | 1.1 | 3.11 | 1 | *E.u.* | LG6 |
| 10H11 | EST | small GTP-binding protein | GTP binding protein | AJ627797 | F:GGGCTCATGGGATCATTGTC<br>R:GCTCGCCATCCTGTTCTTG | 91 | 1.1 | 0.54 | 1 | *E.g.* | LG6 |
| 06D03 | EST | 60S Ribosomal protein L7 | Protein synthesis | AJ627665 | F:TCAGGGAAGCAAATAACT<br>R:GCCTCAAAATAGCAAAAC | 90 | 1.1 | 0.71 | 1 | *E.u.* | LG1 |
| 09D03 | EST | subtilisin-like proteinase | Proteolysis | AJ627753 | F:GAAGGATGGGGTTCACAAGG<br>R:CAACATGGATCAACCGAAAA | 168 | 1.1 | 0.02 | 1 | *E.u.* | LG7 |
| 06A07 | EST | Dehydrin | Stress Response | AJ627650 | F:AGACGAAGCAGGAGGAGGTGAT<br>R:GAAACAGGCCAAAACTAATACA | 90 | 1.1.1.1 | 6.09 | 3 | *E.u. E.g.* | LG1 |
| 11B10 | EST | ceo protein | Defense | AJ627804 | F:GAGGAGGAGCAGAAAGCAGG<br>R:AAATCAGCATGAAAGCGGTA | 176 | 1.1.1.1 | 4.77 | 3 | *E.u. E.g.* | LG9 |
| 08E02 | EST | putative phytochelatin synthetase | Detoxification | AJ627733 | F:TCAAAAGGACAAGAACACG<br>R:ACAGGCAGTAACTTCCGAC | 90 | 1.1 | 0.4 | 1 | *E.g.* | LG6 |
| GF86 | EST | alpha-tubulin | Cell Wall | n.a. | F:CTTCCACCCTGAGCAACTC<br>R:AAAGCACAGCAACATCGGT | 173 | 1.1.1.1 | 0.34 | 3 | *E.u. E.g.* | LG2 |
| EgTUBA1 | STS | alpha Tubulin | Cell Wall | U37794 | Gion *et al.* 2000 | 175 | 1.1.1.1 | 0.06 | 3 | *E.u. E.g.* | LG2 |
| CHS | STS | chalcone synthase | Flavonoide Biosynthesis | X94995,X60754,X58339,X94706 | F:GCAGTCACGTTTCGTGGC<br>R:TTGAAATGAGCCCAGGAAC | 89 | 1.1.1.1 | 6.69 | 3 | *E.u. E.g.* | LG8 |
| 00A00 | EST | propionyl-CoA carboxylase | Lipid metabolism | n.a. | unpublished | 173 | 1.1 | 0.01 | 1 | *E.u.* | LG4 |
| SAH | STS | adenosylhomocysteinase | Cell Wall | n.a. | Kirst *et al* . 2004 | 84 | 1.1 | 0.76 | 1 | *E.u.* | LG3 |
| ELF1 | STS | LEAFY/FLORICAULA (LFY1) | Regulation | AFO56550, AFO34806 | F:ATCAAYGCWRYIYTVAATGA<br>R:GCRGCVACATTGATGTTITC | 194 | 1.1 | 0.33 | 1 | *E.u.* | LG4 |
| ELF2 | STS | LEAFY/FLORICAULA (LFY1) | Regulation | AFO56550, AFO34806 | F:ATCAAYGCWRYIYTVAATGA<br>R:GCRGCVACATTGATGTTITC | 193 | 1.1 | 5.64* | 1 | *E.u.* | LG4 |
| EgHypar | STS | auxin-induced glutathione-S-transferases | Regulation | U80615 | Gion *et al.* 2000 | 181 | 1.1 | 0.67 | 1 | *E.u.* | LG7 |
| 08E12 | EST | unknown | - | AJ627737 | F:TTCAATAATCCCTGGTCAAT<br>R:CCAAACGCACCTTAATAGTC | 170 | 1.1.1.1 | 9.65 | 7 | *E.u. E.g.* | LG5 |
| 06D01 | EST | unknown | - | n.a. | F:TGTTGGCTGGCATTTCCTTCC<br>R:CCTCTGGGCATTCACCTACCG | 89 | 1.1.1.1 | 0.39 | 3 | *E.u. E.g.* | LG2 |
| 11C08 | EST | unknown | - | AJ627806 | F:AGGCTAAAGTGGTGGGGTG<br>R:AAGTGGAAAGGAGAAAGAGG | 90 | 1.1 | 0.18 | 1 | *E.u.* | LG1 |
| 06D09 | EST | unknown | - | AJ627667 | F:GAAGGCTTAGAGACGGGTTTT<br>R:ACTGGTTGAGGCACTGGTTTT | 64 | 1.1 | 3.06 | 1 | *E.g.* | LG3 |
| 08F09 | EST | unknown | - | AJ627739 | F:GGGCACTTTCAGGCACCACC<br>R:GCATCGCATCGTATCGGGAG | 171 | 1.1 | 0.01 | 1 | *E.g.* | LG4 |
| 08E12A | EST | unknown | - | AJ627737 | F:TTCAATAATCCCTGGTCAAT<br>R:CCAAACGCACCTTAATAGTC | 170 | 1.1 | 0.09 | 1 | *E.g.* | LG1 |
| 11B02 | EST | unknown | - | AJ627801 | F:TGAAGTTCAGCGGCAAGAAG<br>R:TTGGAGGAAACAGCTAACCAAT | 172 | 1.1 | 7.73** | 1 | *E.g.* | LG7 |
| 10D09 | EST | unknown | - | AJ627777 | F:GTACGGGGGAGTAATGACC<br>R:CTGCCTCCCTTTGATACTG | 93 | 1.1 | 0.01 | 1 | *E.g.* | LG11 |

| | | | | | | | | | | | |
|---|---|---|---|---|---|---|---|---|---|---|---|
| 07H10 | EST | no hit | - | AJ627717 | F:GGCATAGTCGATCACATTAG<br>R:AGTCGCCTGAGATAAACTGG | 176 | 1.1.1.1 | 2.41 | 3 | *E.u. E.g.* | LG3 |
| 10H06 | EST | no hit | - | AJ627793 | F:AATTTATTTGTTCGAGGGTG<br>R:CAACGCATTCGTGAGTTAGT | 170 | 1.1.1.1 | 9.29* | 3 | *E.u. E.g.* | LG4 |
| 08C08 | EST | no hit | - | AJ627729 | F:ACTCACCCAACTTCAACCAA<br>R:CGTTTGCCACGATGTATTTT | 173 | 1.1.1.1 | 19.35*** | 3 | *E.u. E.g.* | LG5 |
| 08B02 | EST | no hit | - | AJ627720 | F:ATTTCACGGGTGACCAAAGA<br>R:ATGCGGCTCAAGGTGTATCA | 172 | 1.1.1.1 | 0.42 | 3 | *E.u. E.g.* | LG6 |
| 07B05 | EST | no hit | - | AJ627697 | F:TCAAGGACGAAGATGAAGG<br>R:CGATTTGAGCAGAGAAAAGG | 91 | 1.1.1.1 | 1.72 | 3 | *E.u. E.g.* | LG8 |
| 06C01 | EST | no hit | - | AJ627657 | F:GCTTGATAGCTCCAAAATAAGG<br>R:GTCTTTGCTTCACCACGAAC | 142 | 1.1.1.1 | 2.51 | 3 | *E.u. E.g.* | LG10 |
| 09F08 | EST | no hit | - | AJ627754 | F:AAAGCACAAGGGAGAAGAAA<br>R:CTCAGAATTAGATGCCAAGA | 172 | 1.1 | 1.9 | 1 | *E.u.* | LG1 |
| 07B12 | EST | no hit | - | AJ627701 | F:TACATGCACACCTGATTGGG<br>R:TGATTTTGCGTCGGTCGTAC | 93 | 1.1 | 5.69* | 1 | *E.u.* | LG1 |
| 07B05B | EST | no hit | - | AJ627697 | F:TCAAGGACGAAGATGAAGG<br>R:CGATTTGAGCAGAGAAAAGG | 91 | 1.1 | 3.18 | 1 | *E.u.* | LG1 |
| 08C02 | EST | no hit | - | AJ627724 | F:GAAATACTGCTTCTCCCAAG<br>R:CACAAGATGCCTCAACAATG | 172 | 1.1 | 1.49 | 1 | *E.u.* | LG3 |
| 12F08 | EST | no hit | - | AJ627859 | F:GAAATCGAAGCTCGTTGCTG<br>R:CAGAGTGCAAATGGGGAAGT | 94 | 1.1 | 2.09 | 1 | *E.u.* | LG4 |
| 09H02 | EST | no hit | - | AJ627757 | F:ACTTTGCTTGTGGTTATTTC<br>R:AATTCCTTCCGACTTGACTT | 172 | 1.1 | 3.3 | 1 | *E.u.* | LG8 |
| 06G02 | EST | no hit | - | AJ627681 | F:TGCAGACCTACTAAGATGGG<br>R:TGGATTAGCTGCAACATCG | 91 | 1.1 | 3.97* | 1 | *E.u.* | LG10 |
| 06F04 | EST | no hit | - | AJ627677 | F:CAATTCGGATTGGACTCAAG<br>R:GCCTCAAGAGGTCACACAAG | 92 | 1.1 | 0.70 | 1 | *E.g.* | LG6 |
| 09C05 | EST | no hit | - | AJ627751 | F:GCCGAGCTGATGCAATAACT<br>R:GACCTTCTTGGGTCCTGAAA | 172 | 1.1 | 0.58 | 1 | *E.g.* | LG10 |
| 00C00 | EST | no hit | - | n.a. | unpublished | 89 | 1.1 | 0.55 | 1 | *E.g.* | LG11 |
| 11F09 | EST | no hit | - | AJ627824 | F:GGAGAAGAAAGCGGAGAC<br>R:TAGCGAGGGAGATGATGA | 89 | 1.1 | 2.53 | 1 | *E.g.* | LG11 |
| EMBRA08 | SSR | - | - | n.a. | Brondani *et al.* 1998 | 173 | 1.1.1.1 | 9.17* | 3 | *E.u. E.g.* | LG1 |
| EMBRA15 | SSR | - | - | n.a. | Brondani *et al.* 1998 | 170 | 1.1.1.1 | 2.66 | 3 | *E.u. E.g.* | LG3 |
| EMBRA02 | SSR | - | - | n.a. | Brondani *et al.* 1998 | 175 | 1.1.1.1 | 2.85 | 3 | *E.u. E.g.* | LG4 |
| EMBRA12 | SSR | - | - | n.a. | Brondani *et al.* 1998 | 177 | 1.1.1.1 | 1.46 | 3 | *E.u. E.g.* | LG7 |
| EMBRA09 | SSR | - | - | n.a. | Brondani *et al.* 1998 | 164 | 1.1.1.1 | 11.23* | 3 | *E.u. E.g.* | LG8 |
| EMBRA20 | SSR | - | - | n.a. | Brondani *et al.* 1998 | 170 | 1.1 | 0.85 | 1 | *E.u.* | LG1 |
| EMBRA07 | SSR | - | - | n.a. | Brondani *et al.* 1998 | 171 | 1.1 | 0.71 | 1 | *E.u.* | LG2 |
| EMBRA10 | SSR | - | - | n.a. | Brondani *et al.* 1998 | 169 | 1.1 | 0.49 | 1 | *E.u.* | LG6 |
| EMBRA16 | SSR | - | - | n.a. | Brondani *et al.* 1998 | 178 | 1.1 | 0.56 | 1 | *E.u.* | LG7 |
| EMBRA11 | SSR | - | - | n.a. | Brondani *et al.* 1998 | 176 | 1.1 | 1.11 | 1 | *E.u.* | LG7 |
| EMBRA05 | SSR | - | - | n.a. | Brondani *et al.* 1998 | 178 | 1.1 | 3.8 | 1 | *E.u.* | LG8 |
| EMBRA19 | SSR | - | - | n.a. | Brondani *et al.* 1998 | 165 | 1.1 | 2.67 | 1 | *E.u.* | LG9 |
| EMBRA13 | SSR | - | - | n.a. | Brondani *et al.* 1998 | 91 | 1.1 | 4.85* | 1 | *E.u.* | LG10 |
| U19_944 | SCAR | - | - | n.a. | F:TTTCTCCAACATTTCCAACAC<br>R:CCTTTGCCTTCATCTTCTTCT | 92 | 1.1.1.1 | 3.29 | 3 | *E.u. E.g.* | LG8 |
| X07_396 | SCAR | - | - | n.a. | F:GCTGGCCGAAAGAAGAAA<br>R:ACTAAGGCCCCGCATGGA | 92 | 1.1.1.1 | 2.36 | 3 | *E.u. E.g.* | LG10 |
| Y01_494 | SCAR | - | - | n.a. | F:GTGAGCACCATAAAGCAGGAG<br>R:TAGGTAAGGAGCCATTTTCAG | 90 | 1.1 | 0.04 | 1 | *E.u.* | LG3 |


References cited:

- Goicoechea M, Lacombe E, Legay S, Mihaljevic S, Rech P, Jauneau A, Lapierre C, Pollet B, Verhaegen D, Chaubet-Gigot N, Grima-Pettenati J: **EgMYB2, a new transcriptional activator from *Eucalyptus* xylem, regulates secondary cell wall formation and lignin biosynthesis.** *Plant J* 2005, **43**:553–567
- Gion JM, Rech P, Grima Pettenati J, Verhaegen D, Plomion C: **Mapping candidate genes in *Eucalyptus* with emphasis on lignification genes.** *Mol Breed* 2000, **6**:441-449
- Kirst M, Myburg AA, De León JPG, Kirst ME, Scott J, Sederoff R: **Coordinated Genetic Regulation of Growth and Lignin Revealed by Quantitative Trait Locus Analysis of cDNA Microarray Data in an Interspecific Backcross of *Eucalyptus*.** *Plant Physiol* 2004, **135**:2368-2378
- Brondani RPV, Brondani C, Tarchini R, Grattapaglia D: **Development, characterisation and mapping of microsatellite markers in *Eucalyptus grandis* and *E. urophylla*.** Theor Appl Genet 1998, 97:816-827
