# Supplementary material for: Comprehensive genetic dissection of wood properties in a widely-grown tropical tree: Eucalyptus
Source: BMC Genomics. 2011 Jun 8;12:301. doi: 10.1186/1471-2164-12-301 (PMC3130712; doi:10.1186/1471-2164-12-301)
Supplement: Additional file 2 — Table S2: Characteristics of E. urophylla and E. grandis genetic maps. The total length (in cM) of each linkage group (LG) was obtained using the Kosambi function. The total number of framework markers (Fr markers) and codominant markers (Co markers) are provided. [file 1471-2164-12-301-S2.PDF]

Supplementary Table S2 : Characteristics of *E. urophylla* and *E. grandis* genetic maps. The total map length (in cM) of each linkage group (LG) was obtained using the Kosambi mapping function. The total number of framework markers (Fr markers) and codominant markers (Co markers) are provided.

	E. urophylla			*E. grandis*		
LG	Length (cM)	Fr. markers	Co. markers	Length (cM)	Fr. markers	Co. markers
1	145	10	8	86	8	5
2	124	12	5	107	11	5
3	130	11	6	138	13	4
4	124	8	10	138	9	5
5	147	10	3	85	7	3
6	145	11	5	84	10	6
7	131	16	9	127	10	3
8	114	13	6	102	9	4
9	95	7	5	78	7	2
10	99	7	6	126	13	6
11	129	11	2	145	13	4
Total	1,383	116	65	1,216	110	47
