# Supplementary material for: Comprehensive genetic dissection of wood properties in a widely-grown tropical tree: Eucalyptus
Source: BMC Genomics. 2011 Jun 8;12:301. doi: 10.1186/1471-2164-12-301 (PMC3130712; doi:10.1186/1471-2164-12-301)
Supplement: Additional file 3 — Figure S1: Distribution of WP and growth QTLs on the linkage groups of E. urophylla and E. grandis. [file 1471-2164-12-301-S3.PDF]

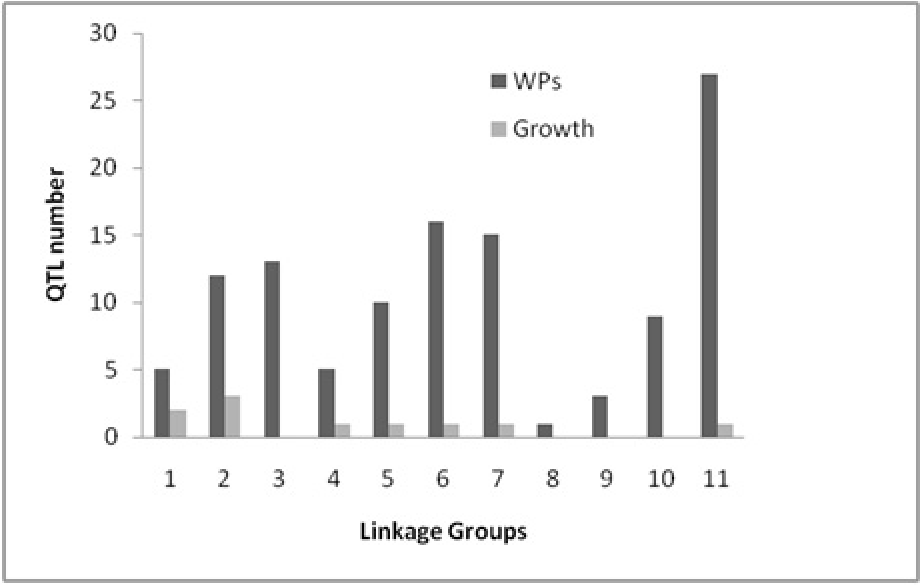


Supplementary figure 1 : Distribution of WP and growth QTLs on the linkage groups of *E. urophylla* and *E. grandis*.
