# Supplementary material for: Comprehensive genetic dissection of wood properties in a widely-grown tropical tree: Eucalyptus
Source: BMC Genomics. 2011 Jun 8;12:301. doi: 10.1186/1471-2164-12-301 (PMC3130712; doi:10.1186/1471-2164-12-301)
Supplement: Additional file 4 — Table S3: Probability of a random co-location of QTL pairs in E. urophylla (E.u) and E. grandis (E.g). [file 1471-2164-12-301-S4.PDF]

Supplementary Table S3: Probability of a random co-location of QTL pairs in *E. urophylla* (*E.u*) and *E. grandis* (*E.g*).

Correlation between traits[a]		Trait ID	Species	Number of intervals (n_1)	Number of co-locations (n_2)	Number of QTLs n_3	n_4	probability (p)	and significance level[b]	Linkage Group (LG)
S	0.61	Pil14 / Pil26	*E. u*	163	3	5	5	0.0001	***	LG3(1+), LG11(2+)
	0.50	Pil14 / Pil38		163	3	5	3	0.0000	***	LG3(1+), LG11(2+)
	0.48	Pil14 / Pil51		163	2	5	5	0.0072	**	LG11(2+)
	0.32	Pil14 / Pil59		163	3	6	5	0.0003	***	LG3(1+), LG11(2+)
	-0.55	Pil14 / E_p		163	1	5	3	0.0875	-	-
	-0.49	Pil14 / E_{2i}		163	2	9	5	0.0238	*	LG2(1-), LG11(1-)
	0.30	Pil14 / Pil59u		163	3	5	5	0.0001	***	LG3(1+), LG11(2+)
	-0.43	Pil14/ µD		163	2	6	5	0.0105	*	LG11(2-)
	0.65	Pil26 / Pil38		163	3	5	3	1E-05	***	LG3(1+), LG11(2+)
	0.66	Pil26 / Pil51		163	2	5	5	0.0072	**	LG11(2+)
	0.48	Pil26 / Pil59		163	1	6	5	0.1622	-	-
	0.40	Pil26 / Pil59u		163	1	5	5	0.1386	-	-
	-0.55	Pil26 / µD		163	2	6	5	0.0105	*	LG11(2-)
	-0.47	Pil26 / E_p		163	1	5	3	0.0875	-	-
	-0.59	Pil26 / E_{30}		163	2	10	5	0.0292	*	LG11(2-)
	-0.51	Pil26 / E_{2i}		163	2	9	5	0.0238	*	LG11(2-)
	0.65	Pil38 / Pil51		163	2	5	3	0.0022	**	LG11(2+)
	0.51	Pil38 / Pil59		163	2	6	3	0.0033	**	LG11(2+)
	0.36	Pil38 / Pil59u		163	1	5	3	0.0875	-	-
	-0.42	Pil38 / µD		163	2	6	3	0.0033	**	LG11(2-)
	-0.32	Pil38 / E_p		163	1	3	3	0.0539	-	-
	-0.39	Pil38 / E_{30}		163	2	10	3	0.0097	**	LG11(1-)
	-0.44	Pil38 / E_{2i}		163	2	9	3	0.0078	**	LG11(1-)
	0.59	Pil51 / Pil59		163	2	6	5	0.0105	*	LG11(2+)
	0.50	Pil51 / Pil59u		163	2	5	5	0.0072	**	LG3(1+), LG11(1+)
	-0.50	Pil51 / µD		163	3	6	5	0.0003	***	LG2(1-), LG11(2-)
	-0.40	Pil51 / E_p		163	1	5	3	0.0875	-	-
	-0.51	Pil51 / E_{30}		163	2	10	5	0.0292	*	LG11(2-)
	-0.52	Pil51 / E_{2i}		163	2	9	5	0.0238	*	LG11(2-)
	0.52	Pil59 / Pil59u		163	1	6	5	0.1622	-	-
	-0.20	Pil59 / E_p		163	1	6	3	0.1037	-	-
	-0.22	Pil59 / E_{2i}		163	1	9	6	0.2563	-	-
	-0.36	Pil59u / µD		163	2	6	5	0.0105	*	LG11(2-)
	-0.34	Pil59u / E_{30}		163	1	10	5	0.2435	-	-
	-0.26	Pil59u / E_{2i}		163	1	9	5	0.225	-	-
	0.58	µD / E_{30}		163	2	10	6	0.0416	*	LG6(1+), LG11(1+)
	0.51	µD / E_{2i}		163	1	9	6	0.2563	-	-
	0.70	E_{30} /E_{2i}		163	5	10	9	3E-05	***	LG2(1+), LG6(1+), LG7(1+), LG11(2+)
	0.88	E_{30} / E_p		163	3	10	3	0.0002	***	LG2(1+), LG7(1+), LG11(1+)
	0.46	E_p / E_{2i}		163	3	9	3	0.0001	***	LG2(1+), LG7(1+), LG11(1+)
	0.24	E_{30} / LGS		163	1	10	5	0.2435	-	-
	0.23	E_{30} / SId		163	1	10	4	0.2065	-	-
	0.29	E_{30} / SIw		163	1	10	1	0.0613	-	-
	0.25	E_{30} / SIm		163	1	10	2	0.1159	-	-
	-0.28	E_{30} / FW		163	1	10	1	0.0613	-	-
	0.24	E_{2i} / SIm		163	1	9	2	0.105	-	-
	-0.21	E_{30} / Klas		163	2	10	6	0.0416	*	LG6(1-)
	0.36	LGS / SIm		163	2	5	2	0.0008	***	LG7(1+), LG10(1+)
	0.34	LGS / SId		163	2	5	4	0.0044	**	LG7(1+), LG10(1+)
	0.41	LGS / SIw		163	1	5	1	0.0307	*	LG7(1+)
	0.74	SId / SIw		163	1	4	1	0.0245	*	LG7(1+)
	0.38	SId / SIm		163	2	4	2	0.0005	***	LG7(1+), LG10(1+)
	0.60	SIw / SIm		163	1	2	1	0.0123	*	LG7(1+)
	0.34	Klas / FW		163	1	6	1	0.0368	*	LG6(1+)
	-0.29	Klas / S/G		163	1	6	5	0.1622	-	-
	0.61	Pil14 / Pil26	*E. g*	127	1	5	4	0.1428	-	-
	0.65	Pil38 / Pil51		127	1	2	2	0.0312	*	LG6(1+)
	0.95	FW / Curl		127	1	3	1	0.0236	*	LG7(1+)
NS	-	LGS / Pil26	*E. u*	163	1	5	5	0.1386	-	-
	-	LGS / E_{2i}		163	1	9	5	0.225	-	-
	-	E_{2i} / FW		163	1	9	1	0.0552	-	-
	-	E_{2i} / SId		163	1	9	4	0.1895	-	-
	-	E_{2i} / SIw		163	1	9	1	0.0552	-	-
	-	E_{2i} / SIm		163	1	9	2	0.105	-	-
	-	LGS / E_p		163	1	5	3	0.0875	-	-
	-	Klas / SId		163	1	6	4	0.1339	-	-
	-	Klas / Pil14		163	1	6	5	0.1622	-	-
	-	Klas / Pil59		163	1	6	6	0.1884	-	-
	-	FW / S/G		163	1	5	1	0.0307	*	LG6(1-)
	-	FWL / Fla		163	1	1	1	0.0061	**	LG2(1+)
	-	µD / Fla		163	1	6	1	0.0368	*	LG2(1+)
	-	Pil51 / Fla		163	1	5	1	0.0307	*	LG2(1-)
	-	G / E_{2i}		163	1	9	3	0.1496	-	-
	-	G / E_{30}		163	1	10	3	0.1641	-	-
	-	G / µD		163	1	6	3	0.1037	-	-
	-	G / Pil59u		163	1	5	3	0.0875	-	-
	-	G / Pil59		163	1	6	3	0.1037	-	-
	-	G / Pil51		163	1	3	3	0.0539	-	-
	-	G / Pil26		163	1	5	3	0.0875	-	-
	-	G / Pil14		163	1	5	3	0.0875	-	-
	-	S/G / Pil14		163	1	5	5	0.1386	-	-
	-	S/G / Pil38	*E. g*	127	1	2	2	0.0312	*	LG6(1+)
	-	S/G / Pil51		127	1	2	2	0.0312	*	LG6(1+)
	-	Pil14 / FW		127	1	4	3	0.09	-	-
	-	Pil26 / FW		127	2	5	3	0.0037	**	LG5(1-)

a/ S : significant, NS: non-significant. Pearson coefficient is given for S correlations.
b/ * 5% ; ** 1% ; *** 0.1% ; - non-significant
