# Supplementary material for: Comprehensive genetic dissection of wood properties in a widely-grown tropical tree: Eucalyptus
Source: BMC Genomics. 2011 Jun 8;12:301. doi: 10.1186/1471-2164-12-301 (PMC3130712; doi:10.1186/1471-2164-12-301)
Supplement: Additional file 5 — Figure S2: Number of detected QTLs as a function of the coefficient of phenotypic variation (CPV) for all the traits. [file 1471-2164-12-301-S5.PDF]

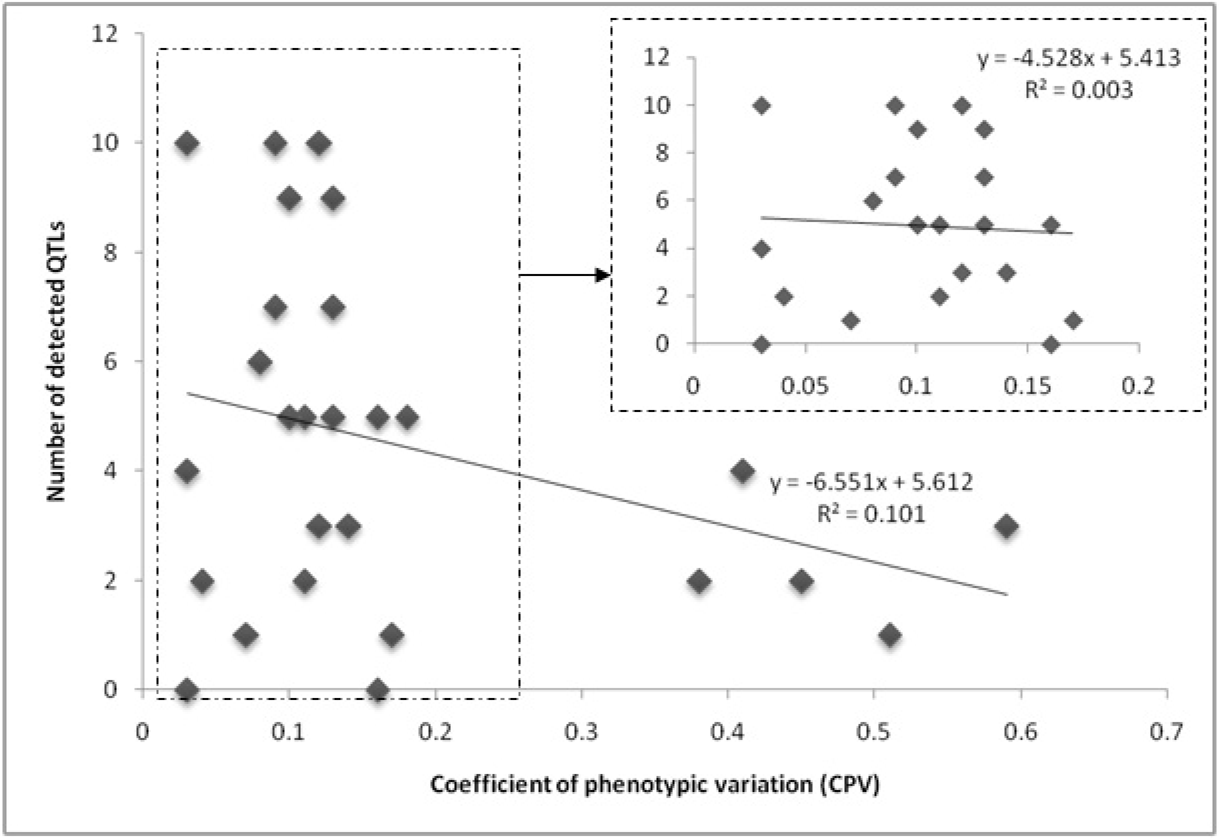

Supplementary Figure 2 : Number of detected QTLs according to coefficient of phenotypic variation (CPV) of the WP traits analysed..
