# Supplementary material for: Comprehensive genetic dissection of wood properties in a widely-grown tropical tree: Eucalyptus
Source: BMC Genomics. 2011 Jun 8;12:301. doi: 10.1186/1471-2164-12-301 (PMC3130712; doi:10.1186/1471-2164-12-301)
Supplement: Additional file 6 — Table S4: Literature review of estimated heritability for wood properties and growth in Eucalyptus species. [file 1471-2164-12-301-S6.PDF]

Supplementary Table S4 : Published results on heritability estimation for wood properties and growth in *Eucalyptus sp* .

Wood property	Trait	h^2 (1)	*Eucalyptus* Species	age	Reference
Mechanical	LGS	0.3 to 0.5	*E. dunnii*	9	Murphy *et al.* (2003)
	Collapse	0.23 to 0.61	*E. nitens*	12	Kube and Raymond (2005)
	Wood basic density	0.42 to 0.75	*E. dunnii*	6.5	Arnold *et al.* (2004)
	Gross Shrinkage	0.25 to 0.31	*E. nitens*	9	Hamilton *et al.* (2009)
	Collapse	0.15 to 0.28	*E. nitens*	9	Hamilton *et al.* (2009)
	Log end-spiltting index	0.31	*E. grandis*	8	dos Santos *et al.* (2004)
Density	Pilodyn	0.3	*E. globulus*	6	Volker *et al.* (2008)
	basic density	0.34	*E. grandis*	8	dos Santos *et al.* (2004)
	Wood density	0.385 to 0.565	*E. pellita*	5.5	Susilawati and Fujisawa (2003)
	basic density	0.51(2)	*E. nitens*	12	Kube *et al.* (2001)
	basic density	0.24	*E. globulus*	13	Poke *et al.* (2006)
	wood density	0.48	*E. urophylla*	NA	Kien *et al.* (2009)
	Basic density	0.37 to 0.42	*E. nitens*	9	Hamilton *et al.* (2009)
	BD	0.44	*E. globulus*	NA	Apialoza *et al.* (2005)
Chemical	acid-soluble lignin content	0.51	*E. globulus*	13	Poke *et al.* (2006)
	extractives content	0.35	*E. globulus*	13	Poke *et al.* (2006)
	cellulose content	0.54	*E. nitens*	12	Kube *et al.* (2001)
	Klason	0.13	*E. globulus*	13	Poke *et al.* (2006)
	cellulose content	0.50	*E. urophylla*	NA	Kien *et al.* (2009)
	cellulose(%)	0.34 to 0.51	*E. nitens*	9	Hamilton *et al.* (2009)
	Cellulose Content	0.84	*E. globulus*	NA	Apialoza *et al.* (2005)
Fibre	FL	0.16	*E. globulus*	NA	Apialoza *et al.* (2005)
	MFA	0.27	*E. globulus*	NA	Apialoza *et al.* (2005)
	microfibril angle	0.293	*E.spp*	8	Lima *et al.* (2004)
	fibre length	0.155 to 0.569	*E. pellita*	5.5	Susilawati and Fujisawa (2003)
	fibre length	0.46	*E. nitens*	12	Kube *et al.* (2001)
	fibre coarseness	0.07	*E. nitens*	12	Kube et al. (2001)
Growth	Diameter	0	*E. globulus*	13	Poke *et al.* (2006)
	Diameter at breast height	0.32	*E. urophylla*	9	Kien *et al.* (2009)
	height	0.14	*E. urophylla*	9	Kien *et al.* (2009)
	DBH	0.19 to 0.34	*E. nitens*	9	Hamilton et al. (2009)
	DBH	0.20	*E. globulus*	NA	Apialoza *et al.* (2005)
	DBH	0.39	*E. nitens*	12	Kube *et al.* (2001)
	Log volume under bark	0.10	*E. grandis*	8	dos Santos *et al.* (2004)
	Diameter	0.24	*E. globulus*	6	Volker *et al.* (2008)

(1): narrow sense heritability
(2): broad sense heritability

References cited:

- Murphy TN, Henson M, Vanclay JK: **Growth stress in Eucalyptus dunnii**. Australian Forestry 2005, 68:144-149
- Kube PD, Raymond CA: **Breeding to minimise the effects of collapse in *Eucalyptus nitens* sawn timber.** *Forest Genetics* 2005, **12**:23-34
- Arnold RJ, Johnson IG, Owen JV: **Genetic variation in growth, stem straightness and wood properties in *Eucalyptus dunnii* trials in Northern New South Wales.** *Forest Genetics* 2004, **11**:1-12
- Hamilton MG, Raymond CA, Harwood CE, Potts BM: **Genetic variation in *Eucalyptus nitens* pulpwood and wood shrinkage traits.** *Tree Genetics & Genomes* 2009, **5**:307-316
- dos Santos PET, Geraldi IO, Garcia JN: **Estimates of genetic parameters of wood traits for sawn timber production in *Eucalyptus grandis*.** *Genet Mol Biol* 2004, **27**:567-573
- Volker PW, Potts BM, Borolo NMG: **Genetic parameters of intra- and inter-specific hybrids of *Eucalyptus globulus* and *E. nitens*.** *Tree Genetics & Genomes* 2008, 4:445-460
- Susilawati S, Fujisawa Y: **Family variation on wood density and fiber length of *Eucalyptus pellita* in seedling seed orchard Pleihari, South Kalimantan. Advances in genetic improvement of tropical tree species.** In Proceedings of the International Conference, Yogyakarta, Indonesia, 1-3 October 2002, 2003:53-56
- Kube PD, Raymond CA, Banham PW: **Genetic parameters for diameter, basic density, cellulose content and fibre properties for *Eucalyptu*s.** *Forest Genetics* 2001, **8**:285-294
- Poke FS, Potts BM, Vaillancourt RE, Raymond CA: **Genetic parameters for lignin, extractives and decay in *Eucalyptus globulus*.** *Ann For Sci* 2006, **63**:813-821
- Kien ND, Quang TH, Jansson G; Harwood C, Clapham D, Arnold S: **Cellulose content as a selection trait in breeding for kraft pulp yield in *Eucalyptus urophylla*.** *Ann For Sci* 2009, **66**:7111-7118
- Apiolaza LA, Raymond CA, Yeo BJ: **Genetic variation of physical and chemical wood properties of *Eucalyptus globulus*.** *Silvae Genet* 2005, **54**:160-166
- Lima JT, Breese MC, Cahalan CM : **Variation in microfibril angle in *Eucalyptus* clones.** *Holzforschung* 2004, **58**:160-166
