# Supplementary material for: Comprehensive genetic dissection of wood properties in a widely-grown tropical tree: Eucalyptus
Source: BMC Genomics. 2011 Jun 8;12:301. doi: 10.1186/1471-2164-12-301 (PMC3130712; doi:10.1186/1471-2164-12-301)
Supplement: Additional file 7 — Table S5: Correspondences between linkage groups (LGs) of this study and that of Brondani et al. [50] based on SSR markers. [file 1471-2164-12-301-S7.PDF]

Supplementary Table S5: Correspondences between linkage groups (LGs) of this study and that of Brondani et al. (1998) based on SSR markers.

| EMBRA Markers | Linkage Group ID | | | |
|---|---|---|---|---|
| | This study | | Brondani *et al.* 1998 | |
| | *E. urophylla* | / *E. grandis* | *E. urophylla* | / *E. grandis* |
| EMBRA20 | LG1 | - | - | LG7 |
| EMBRA8 | LG1 | LG1 | - | LG6 |
| EMBRA7 | LG2 | - | LG9 | - |
| EMBRA15 | LG3 | LG3 | LG8 | LG8 |
| EMBRA2 | LG4 | LG4 | - | LG11 |
| EMBRA10 | LG6 | - | LG10 | LG10 |
| EMBRA12 | LG7 | LG7 | LG1 | LG1 |
| EMBRA16 | LG7 | - | LG1 | LG1 |
| EMBRA11 | LG7 | - | LG1 | LG1 |
| EMBRA5 | LG8 | - | LG5 | LG5 |
| EMBRA9 | LG8 | LG8 | LG5 | LG5 |
| EMBRA19 | LG9 | - | LG4 | LG4 |
| EMBRA13 | LG10 | - | LG9 | LG9 |

Reference cited:

Brondani RPV, Brondani C, Tarchini R, Grattapaglia D: **Development, characterisation and mapping of microsatellite markers in *Eucalyptus grandis* and *E. urophylla*.** *Theor Appl Genet* 1998, **97**:816-827
